# Supplementary figures and images for: Strong static magnetic field delayed the early development of zebrafish
Source: Open Biol. 2019 Oct 30;9(10):190137. doi: 10.1098/rsob.190137 (PMC6833226; doi:10.1098/rsob.190137)

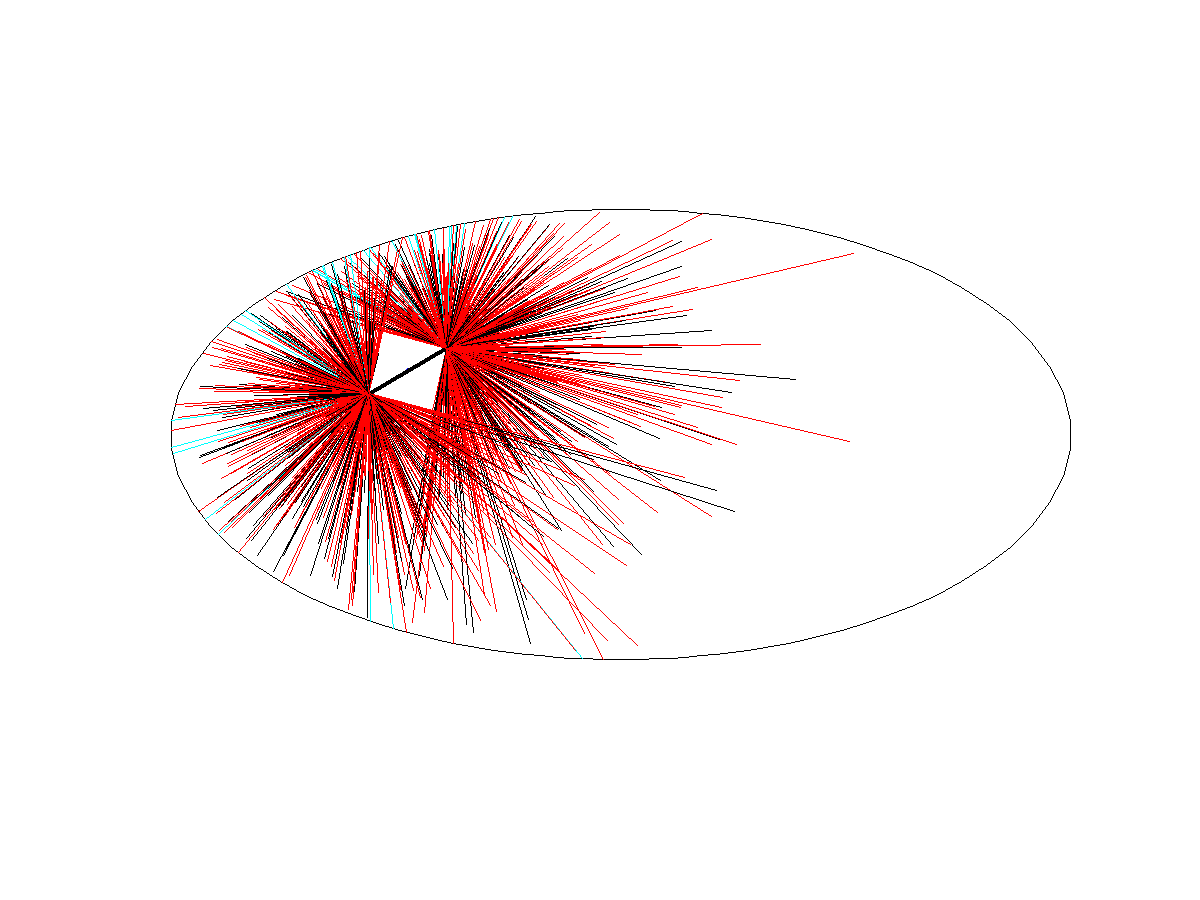

Supplement: Original data [file rsob190137supp1.zip › original data for RSOB-19-0137/Figure 7 theory Matlab modeling simulation original data/result_1/1MTGX_10000.png]

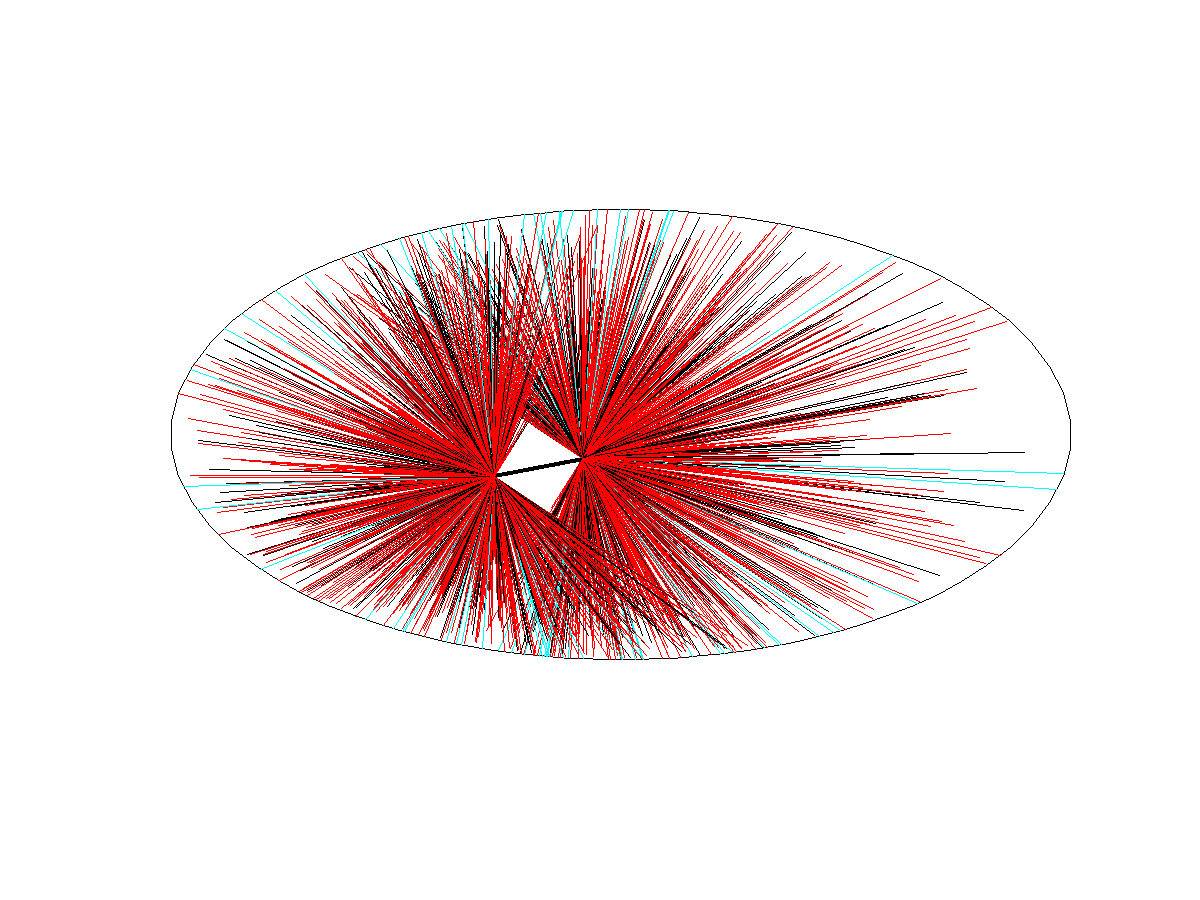

Supplement: Original data [file rsob190137supp1.zip › original data for RSOB-19-0137/Figure 7 theory Matlab modeling simulation original data/result_1/1MTGX_30000.png]

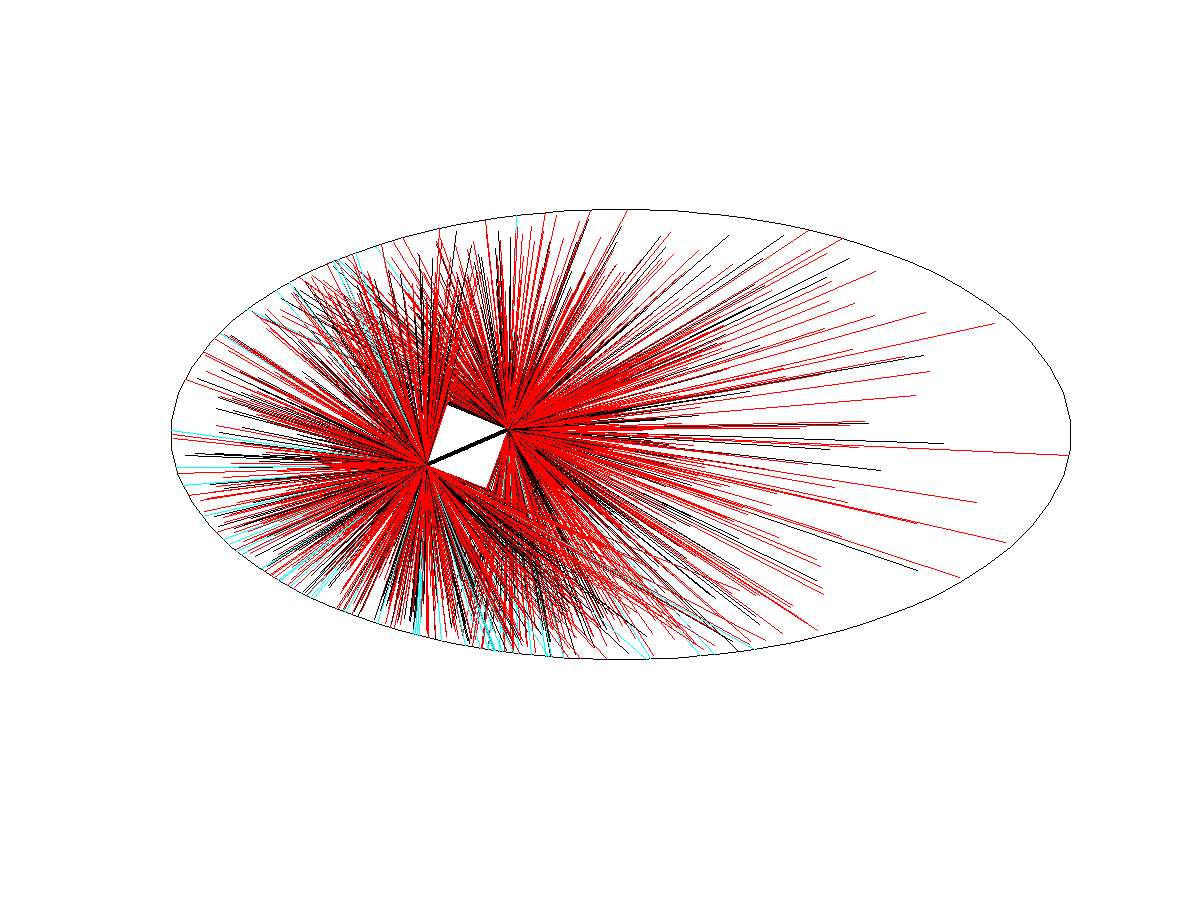

Supplement: Original data [file rsob190137supp1.zip › original data for RSOB-19-0137/Figure 7 theory Matlab modeling simulation original data/result_1/1MTGX_20000.png]

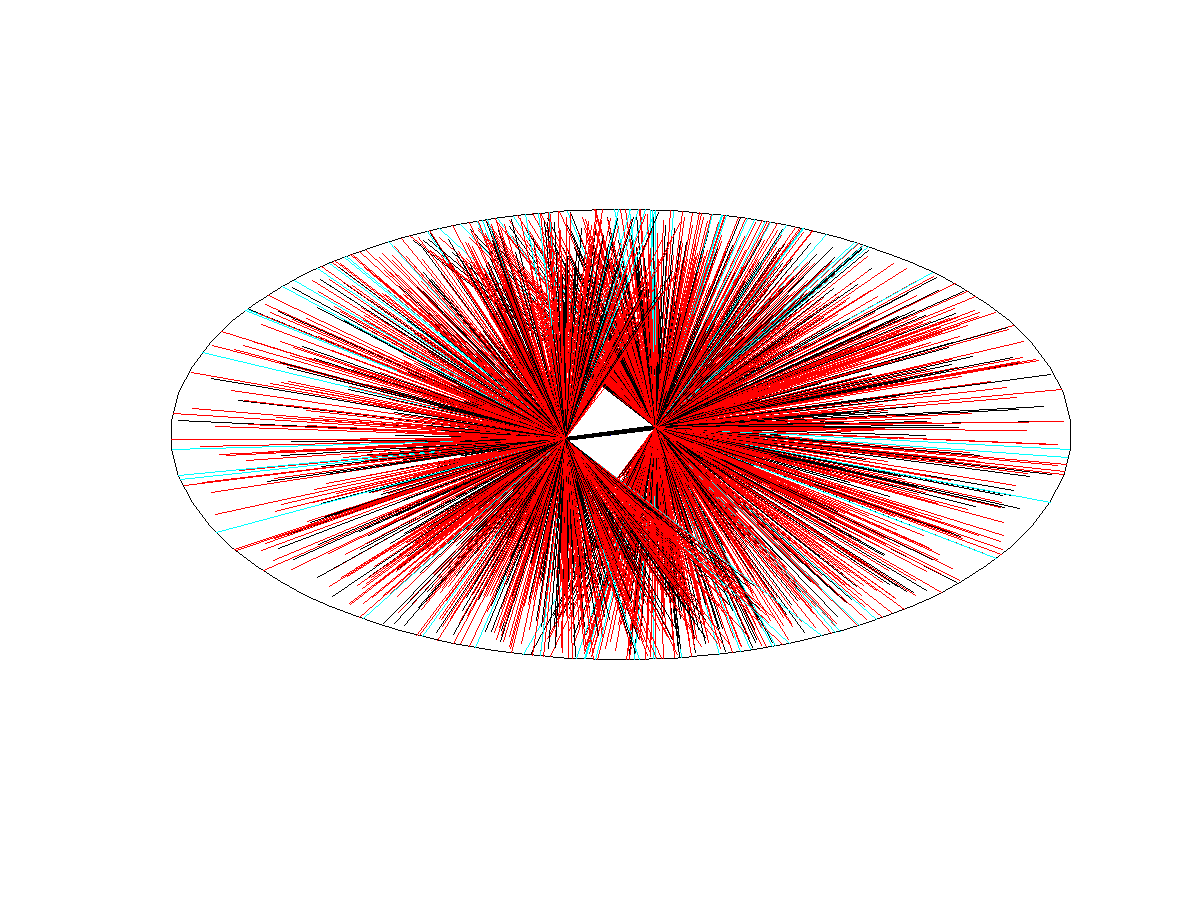

Supplement: Original data [file rsob190137supp1.zip › original data for RSOB-19-0137/Figure 7 theory Matlab modeling simulation original data/result_1/1MTGX_40000.png]

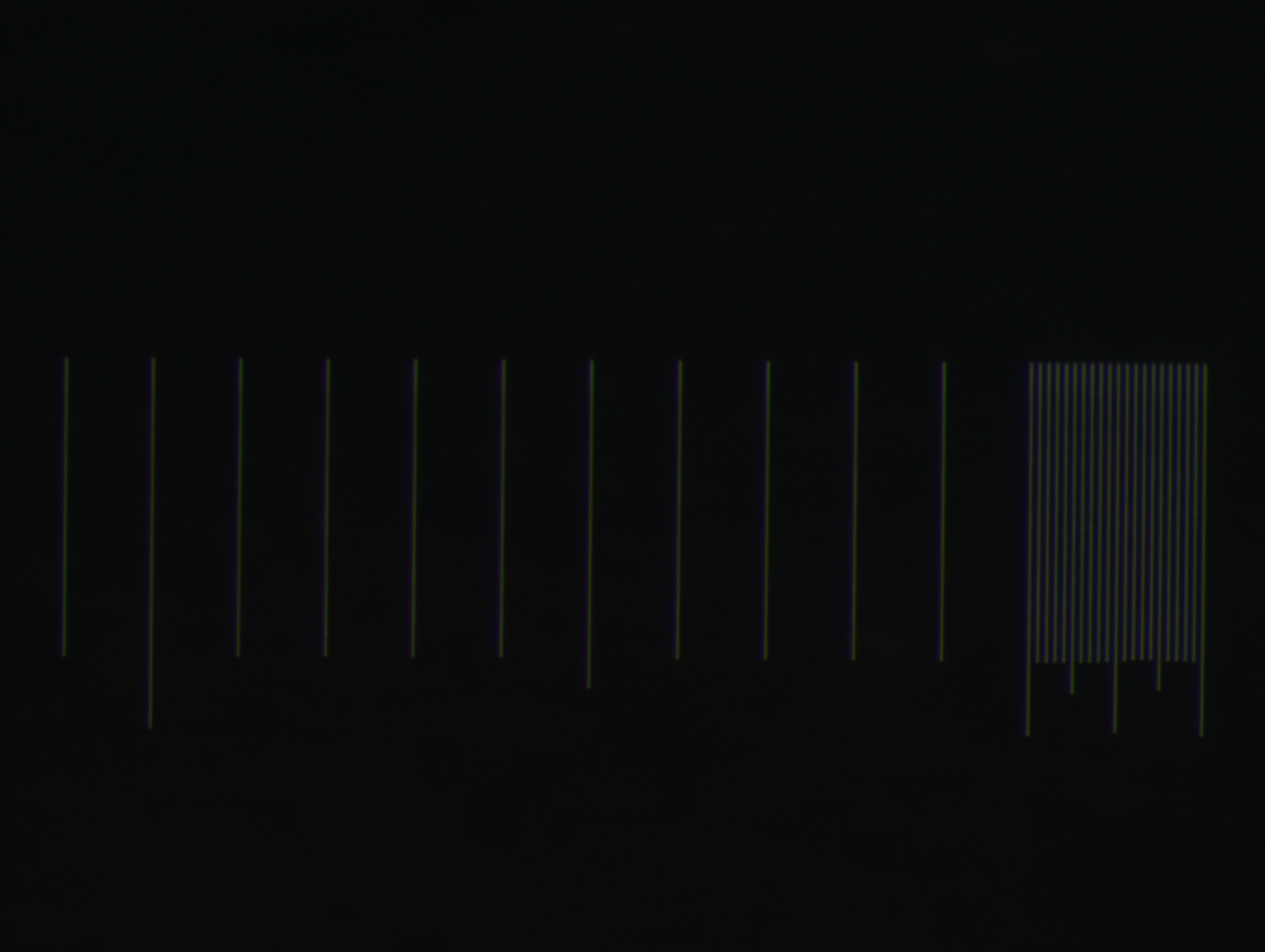

Supplement: Original data [file rsob190137supp1.zip › original data for RSOB-19-0137/Figure 3 Paryngeal arches original data/scales/scale-10X.tif]

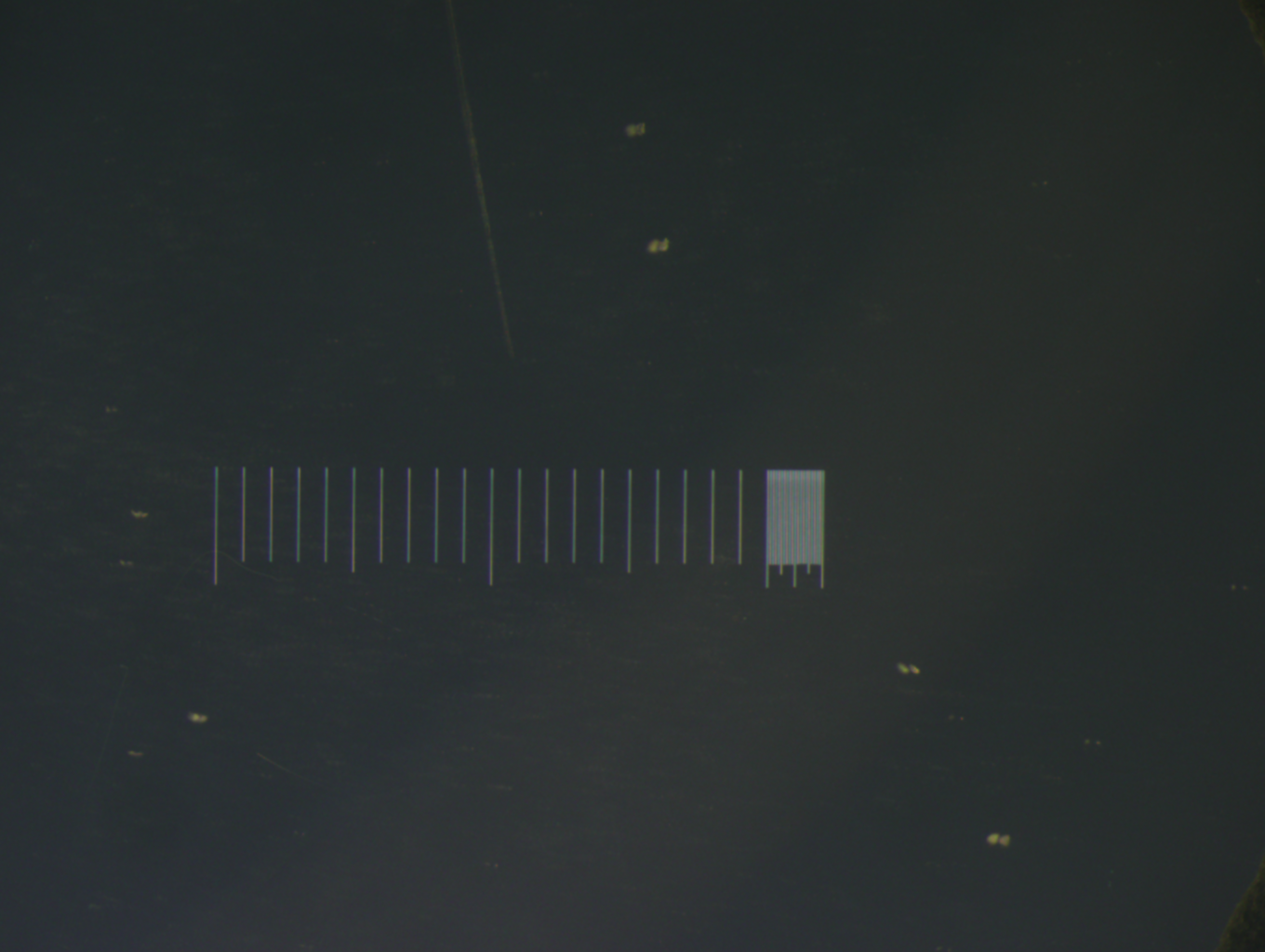

Supplement: Original data [file rsob190137supp1.zip › original data for RSOB-19-0137/Figure 3 Paryngeal arches original data/scales/scale-3.2X.tif]

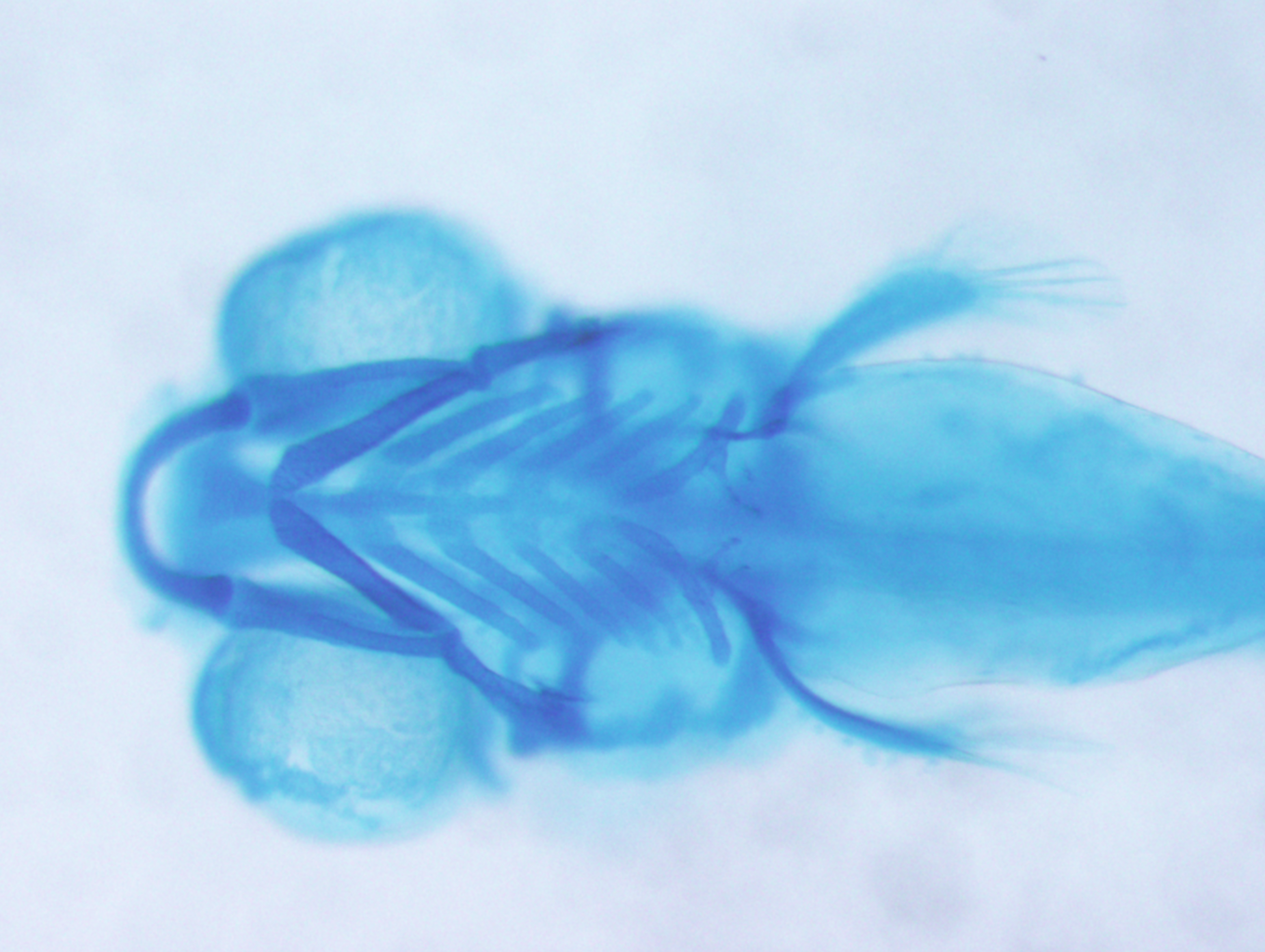

Supplement: Original data [file rsob190137supp1.zip › original data for RSOB-19-0137/Figure 3 Paryngeal arches original data/6 dpf/C6-6-10X.tif]

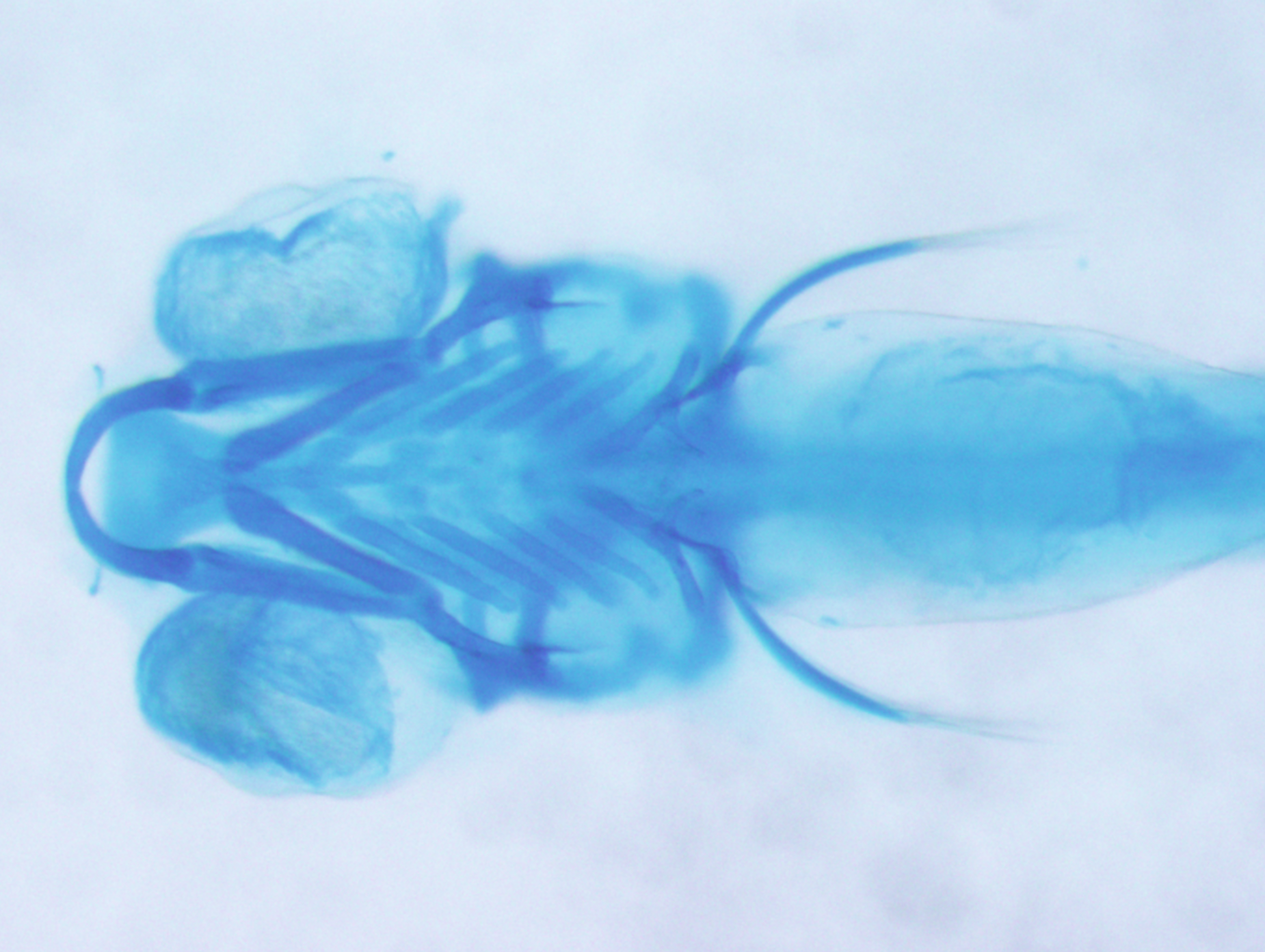

Supplement: Original data [file rsob190137supp1.zip › original data for RSOB-19-0137/Figure 3 Paryngeal arches original data/6 dpf/M6-4-10X.tif]

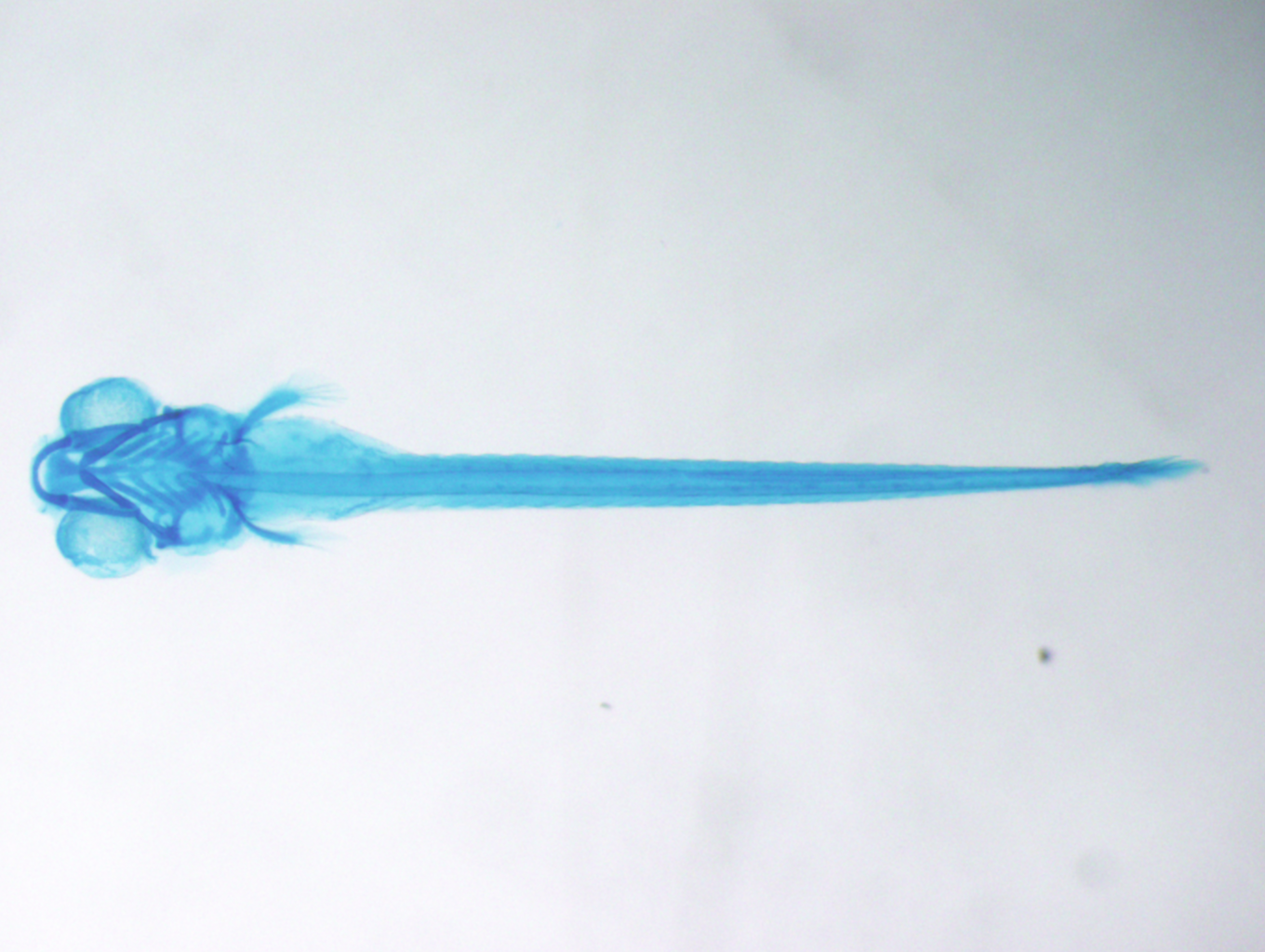

Supplement: Original data [file rsob190137supp1.zip › original data for RSOB-19-0137/Figure 3 Paryngeal arches original data/6 dpf/C6-5-3.2X.tif]

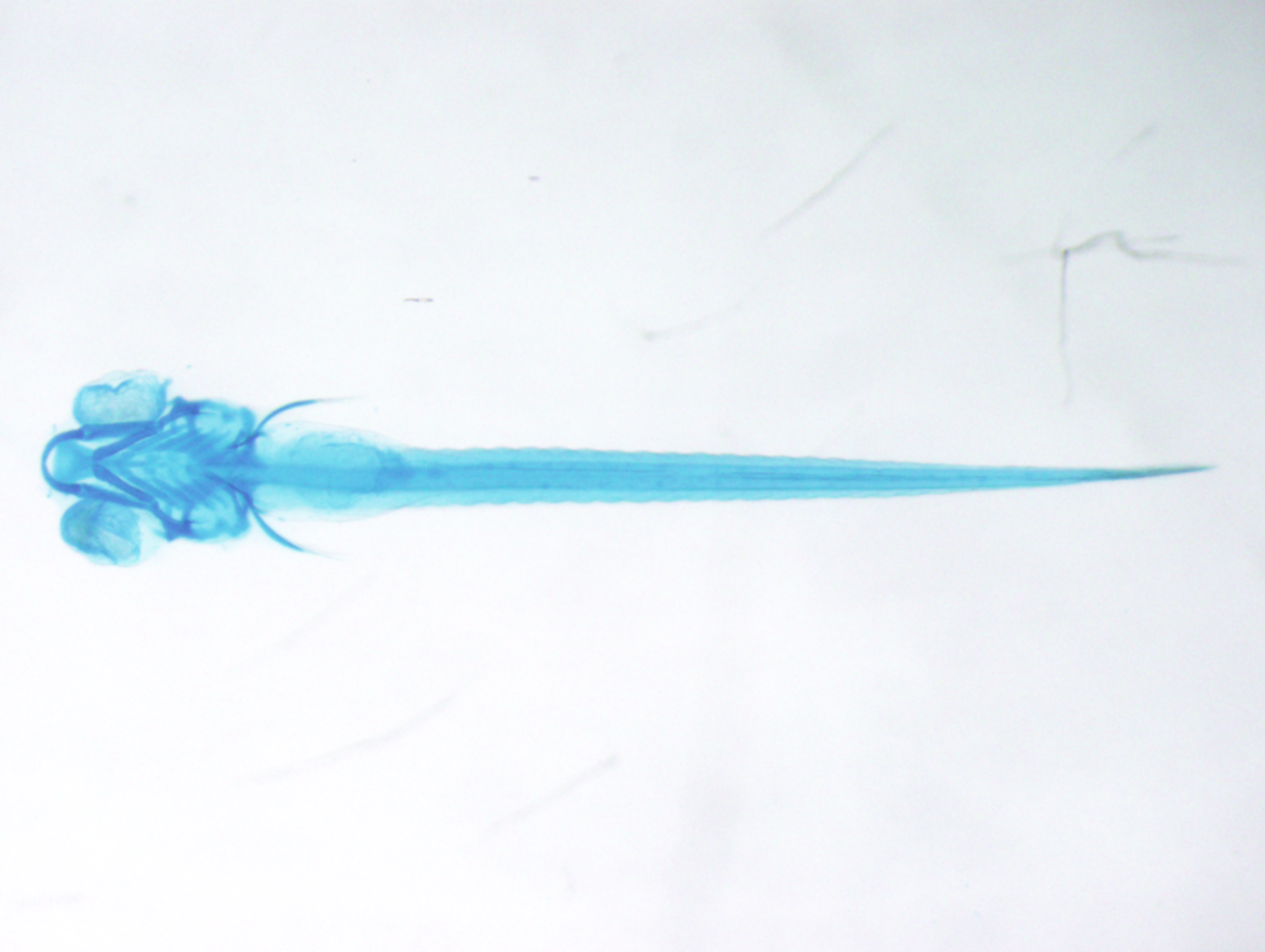

Supplement: Original data [file rsob190137supp1.zip › original data for RSOB-19-0137/Figure 3 Paryngeal arches original data/6 dpf/M6-1-3.2X.tif]

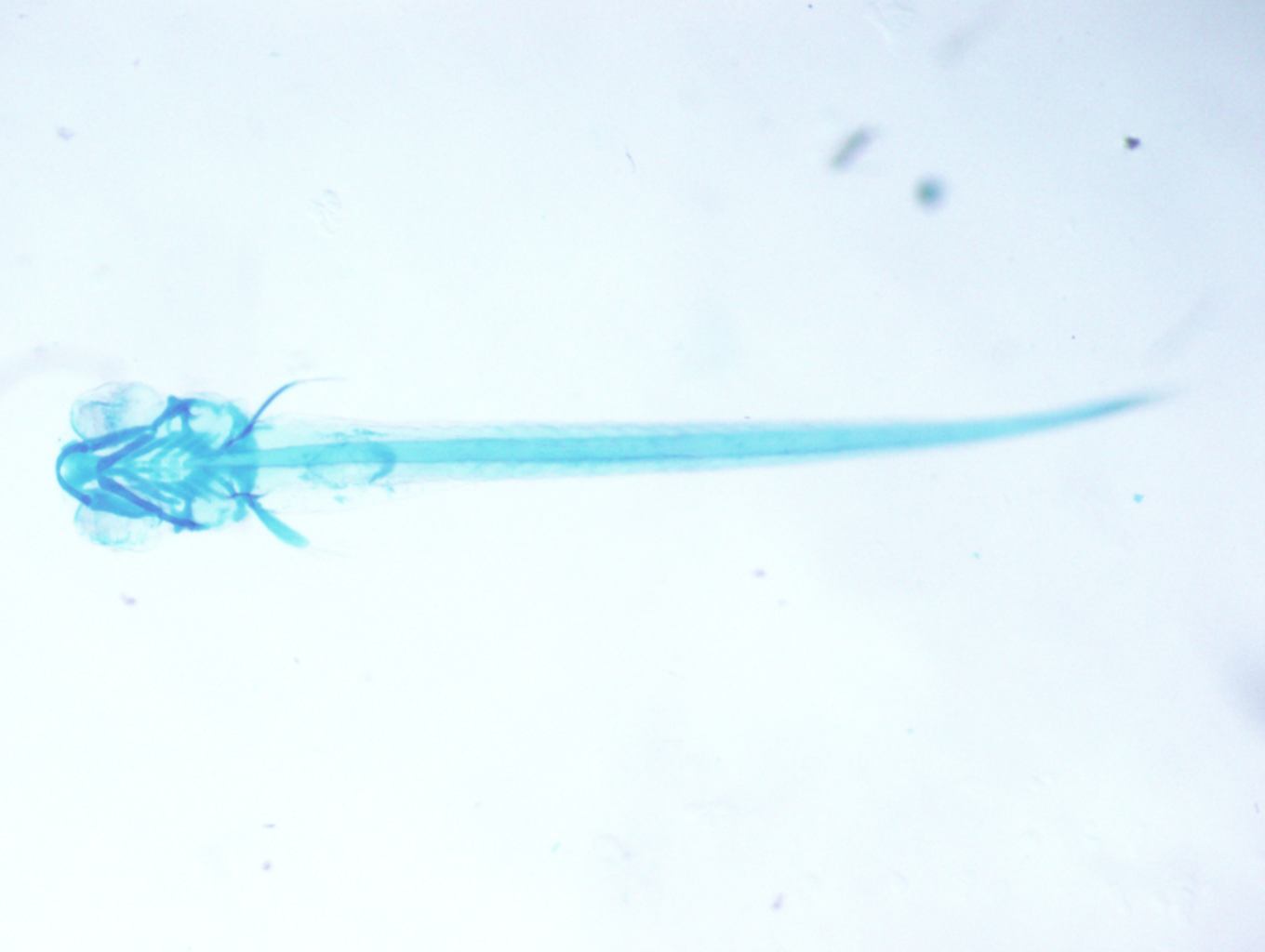

Supplement: Original data [file rsob190137supp1.zip › original data for RSOB-19-0137/Figure 3 Paryngeal arches original data/5 dpf/M5-1-3.2X.tif]

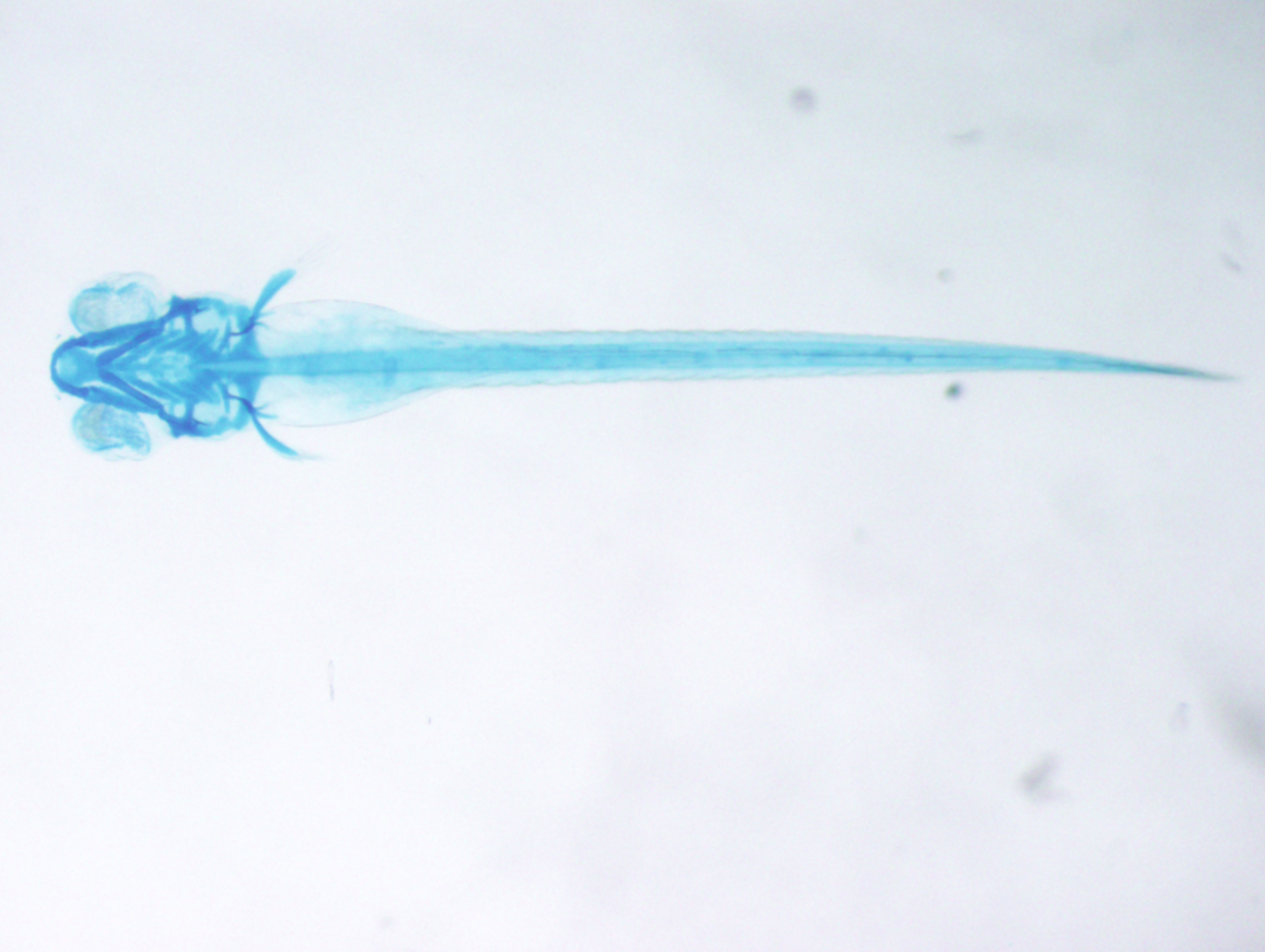

Supplement: Original data [file rsob190137supp1.zip › original data for RSOB-19-0137/Figure 3 Paryngeal arches original data/5 dpf/C5-4-3.2X.tif]

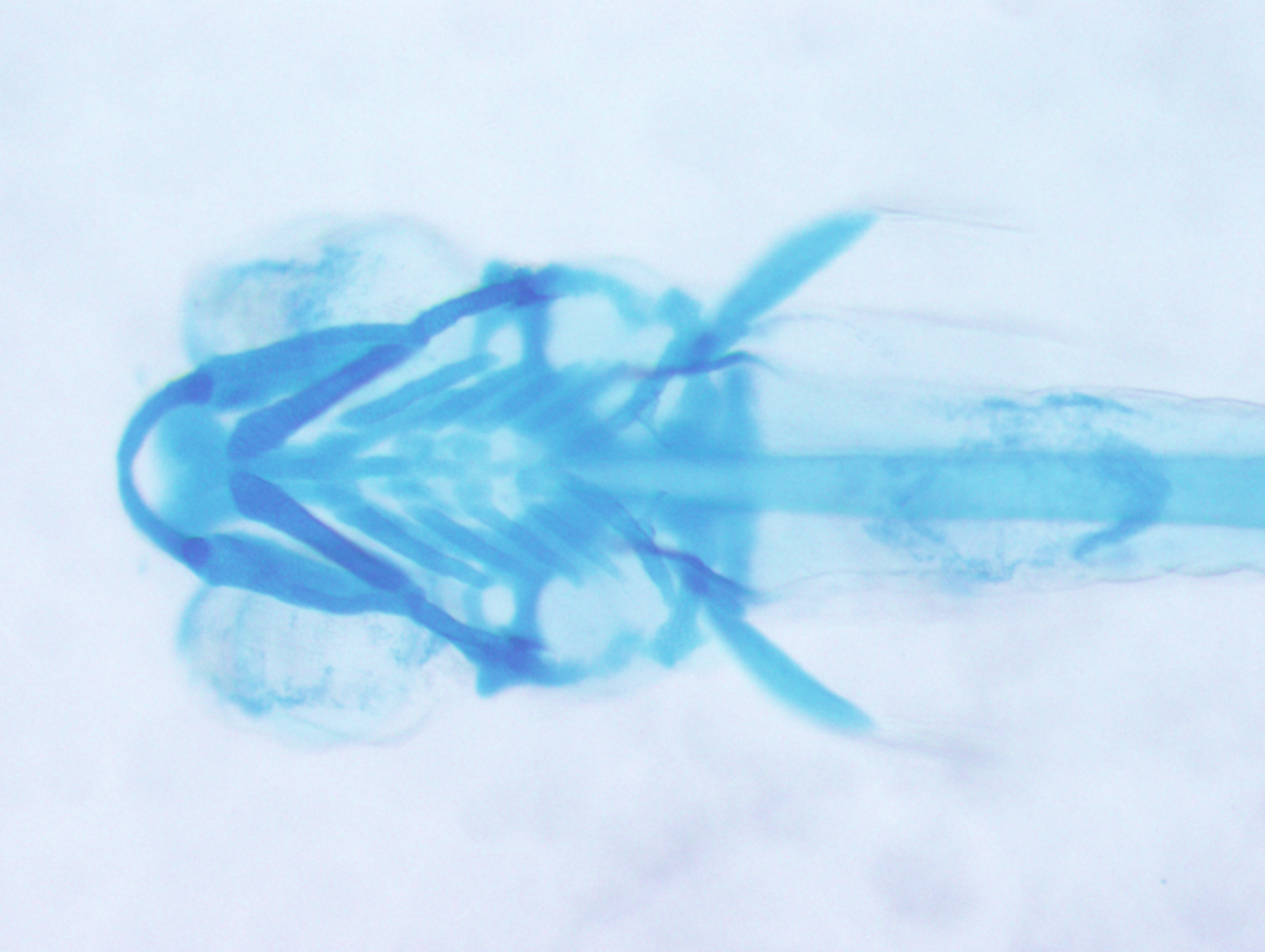

Supplement: Original data [file rsob190137supp1.zip › original data for RSOB-19-0137/Figure 3 Paryngeal arches original data/5 dpf/M5-8-10X.tif]

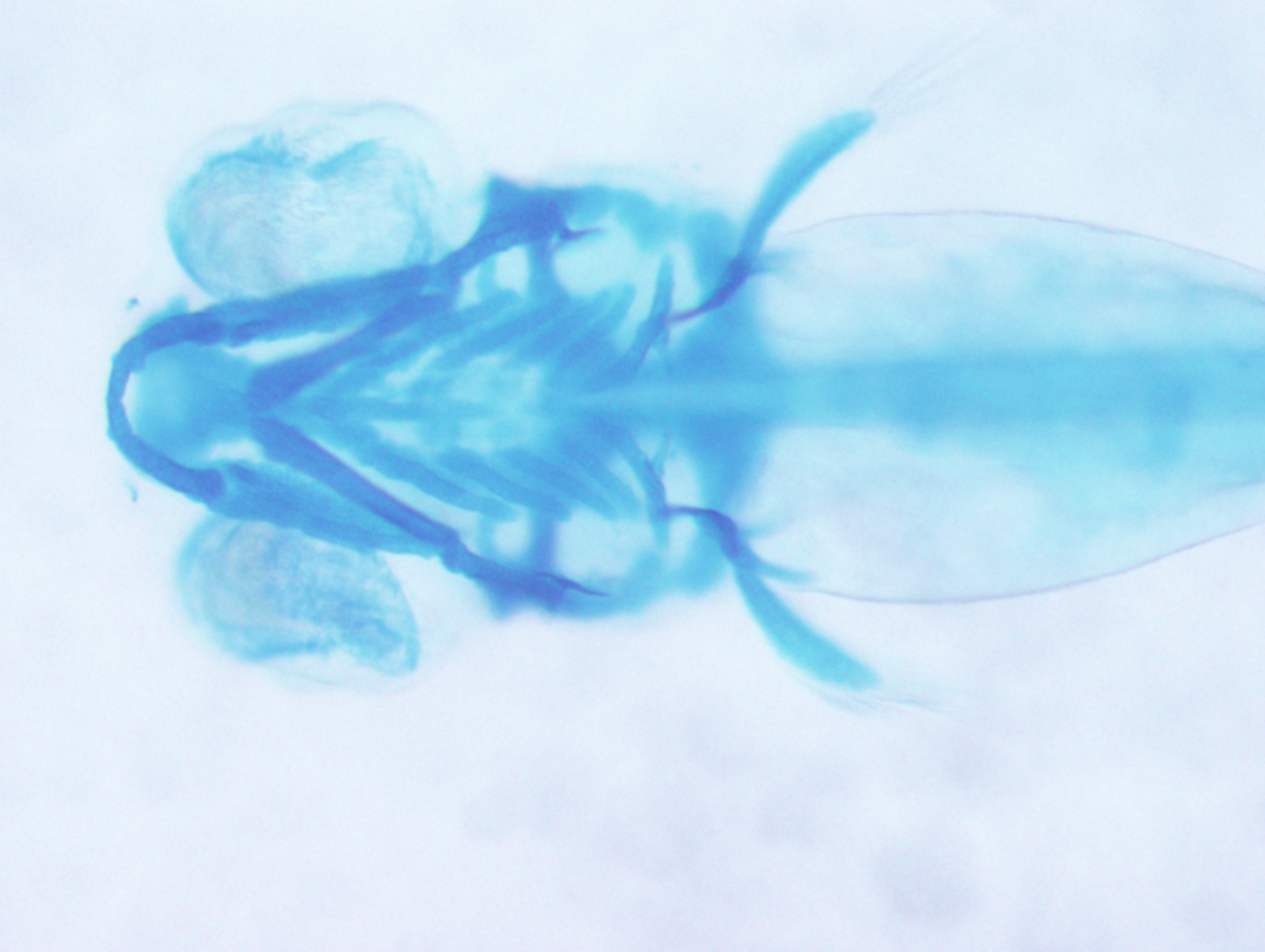

Supplement: Original data [file rsob190137supp1.zip › original data for RSOB-19-0137/Figure 3 Paryngeal arches original data/5 dpf/C5-7-10X.tif]

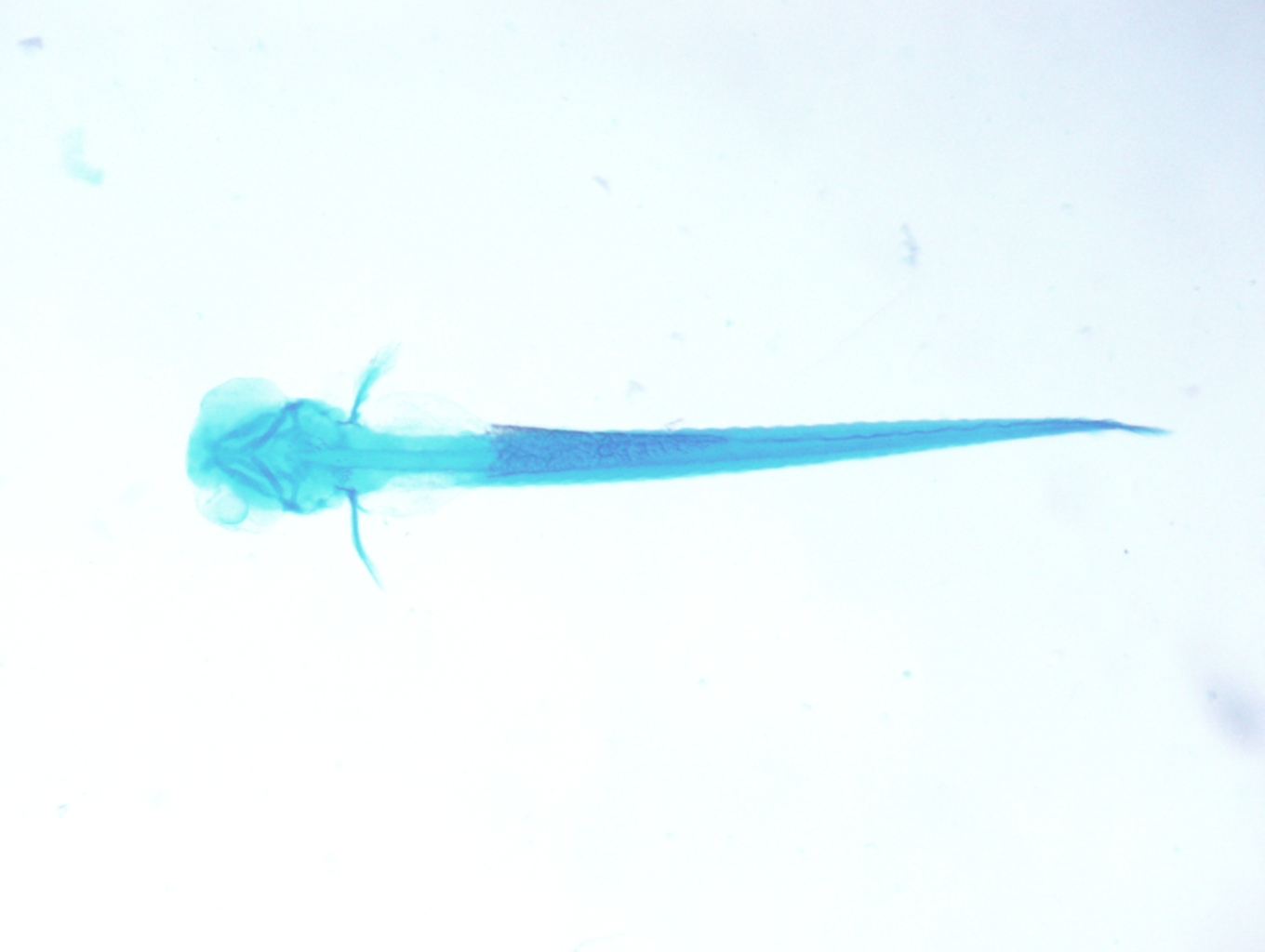

Supplement: Original data [file rsob190137supp1.zip › original data for RSOB-19-0137/Figure 3 Paryngeal arches original data/4 dpf/M4-2-3.2X.tif]

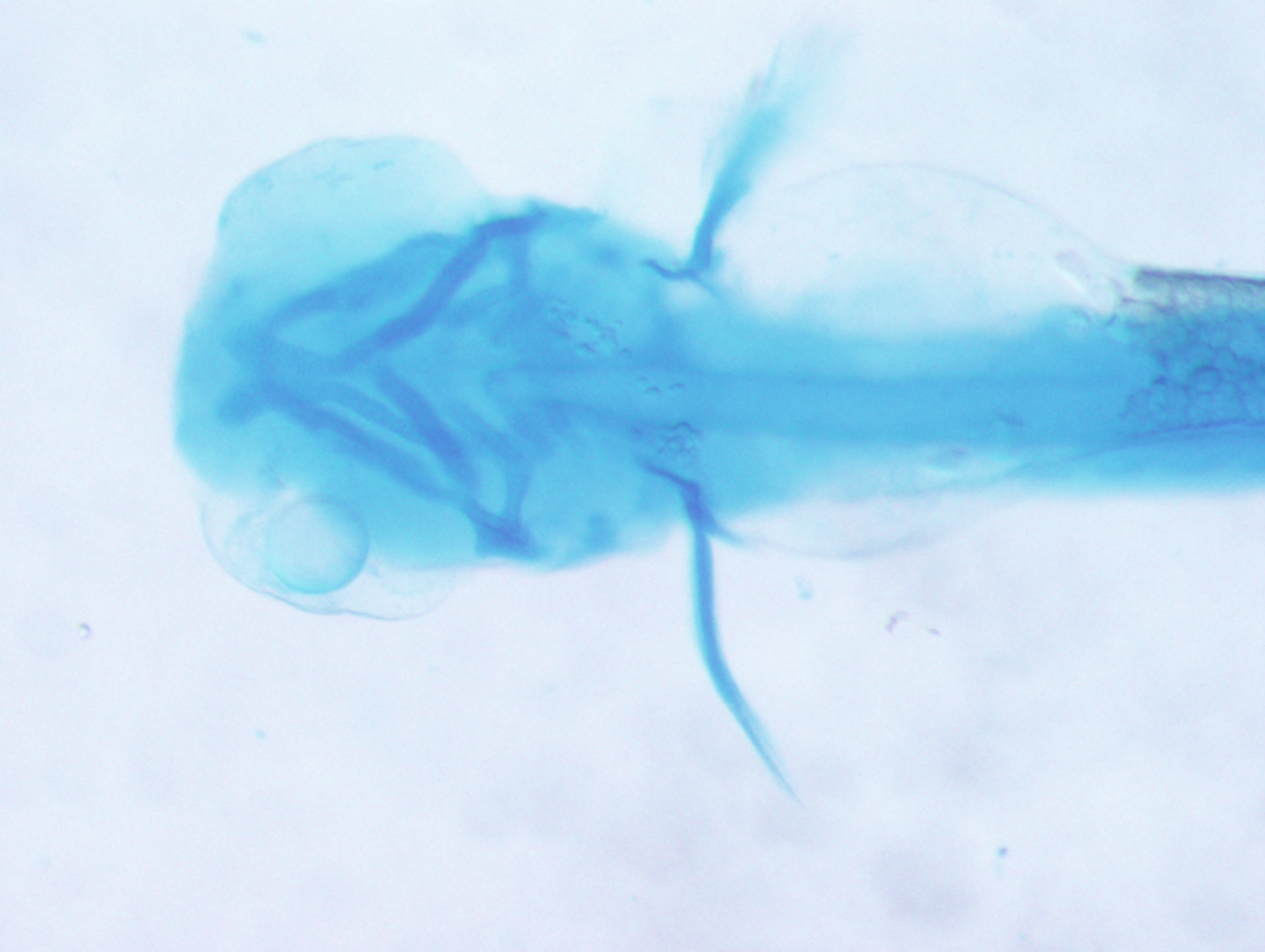

Supplement: Original data [file rsob190137supp1.zip › original data for RSOB-19-0137/Figure 3 Paryngeal arches original data/4 dpf/M4-3-10X.tif]

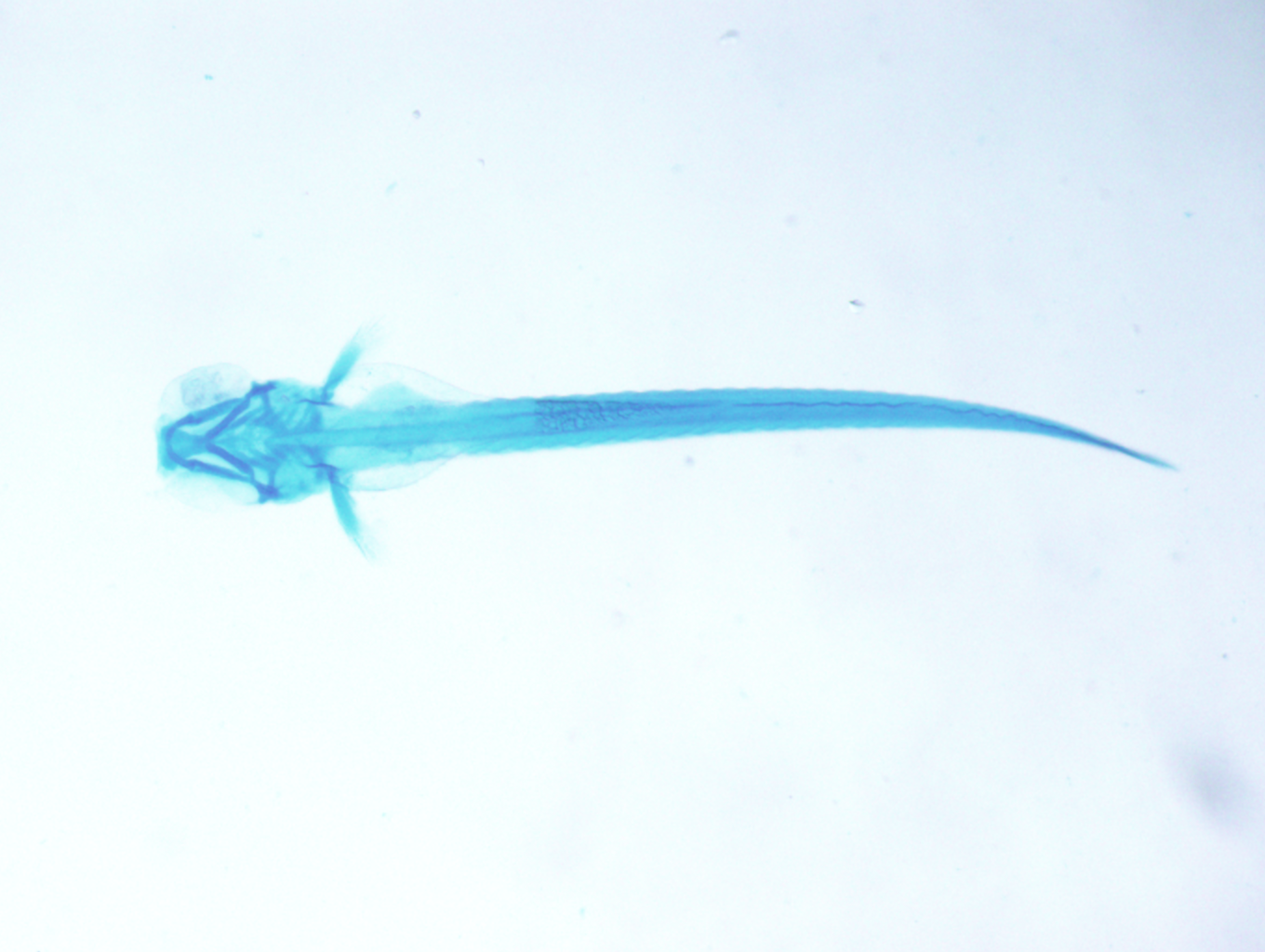

Supplement: Original data [file rsob190137supp1.zip › original data for RSOB-19-0137/Figure 3 Paryngeal arches original data/4 dpf/C4-2-3.2X.tif]

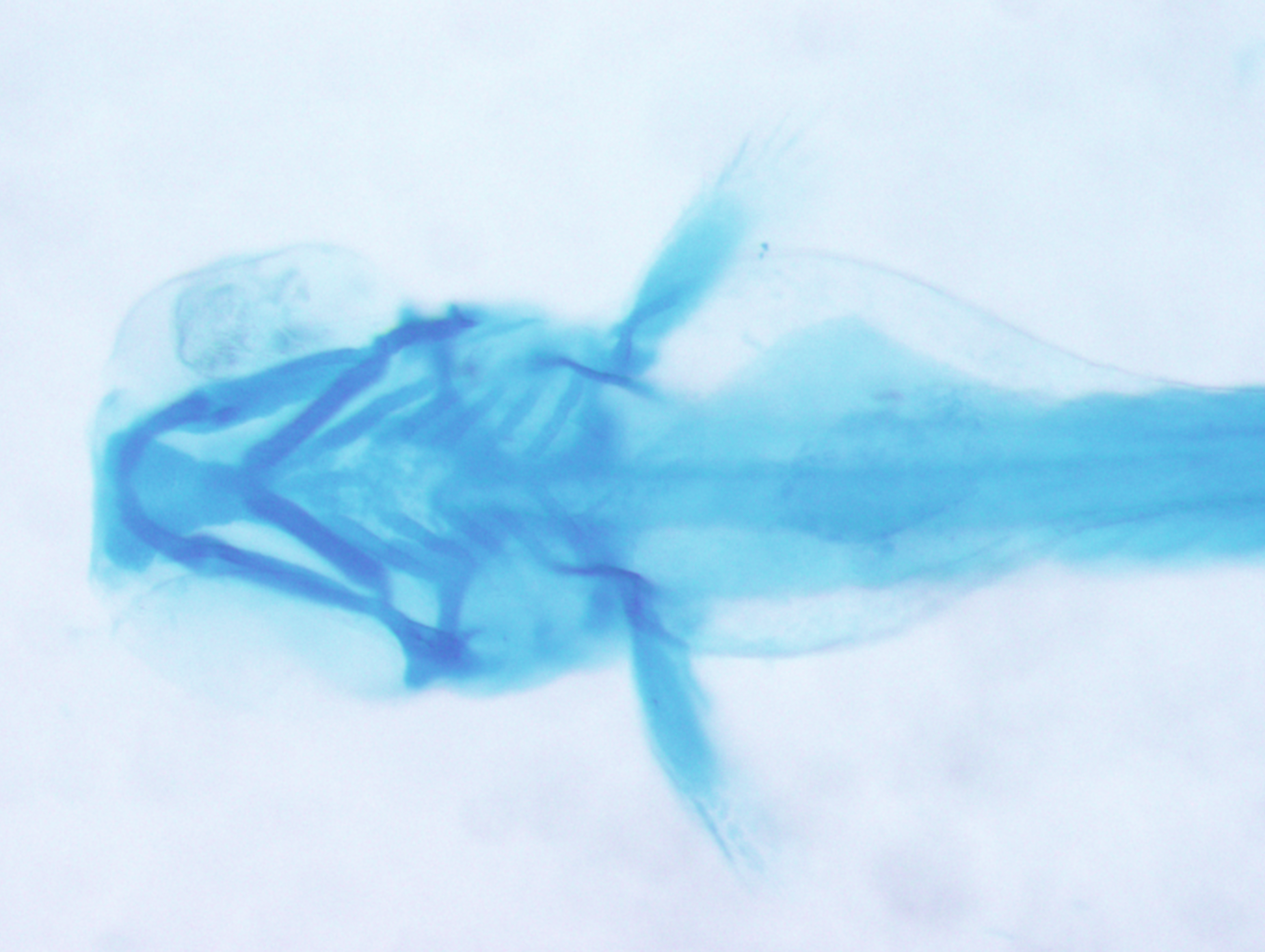

Supplement: Original data [file rsob190137supp1.zip › original data for RSOB-19-0137/Figure 3 Paryngeal arches original data/4 dpf/C4-5-10X.tif]

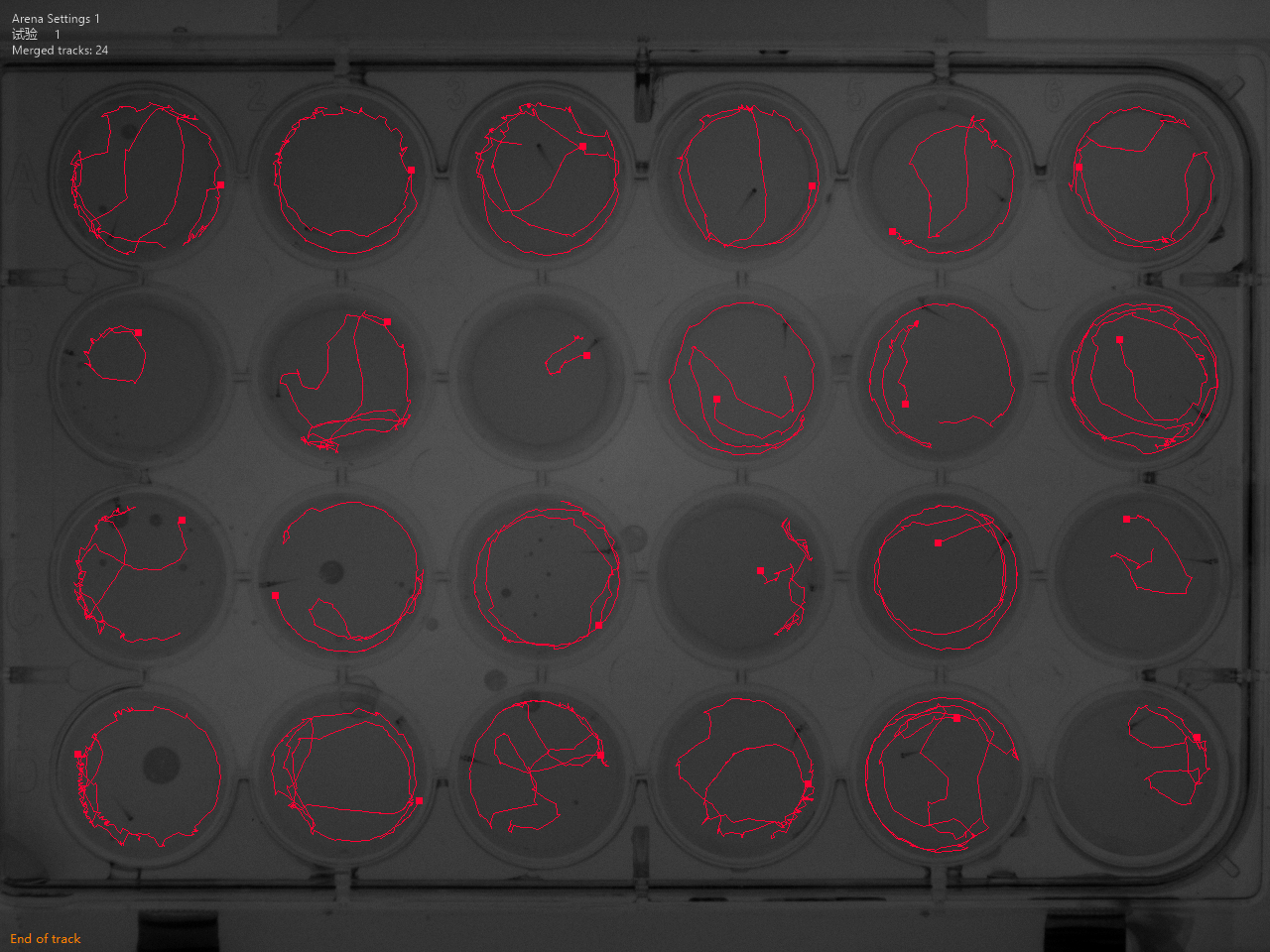

Supplement: Original data [file rsob190137supp1.zip › original data for RSOB-19-0137/Figure 5 behavior original data/Original-Track Visualization Image 0002-The last 60 seconds-batch two 6 dpf 02.png]

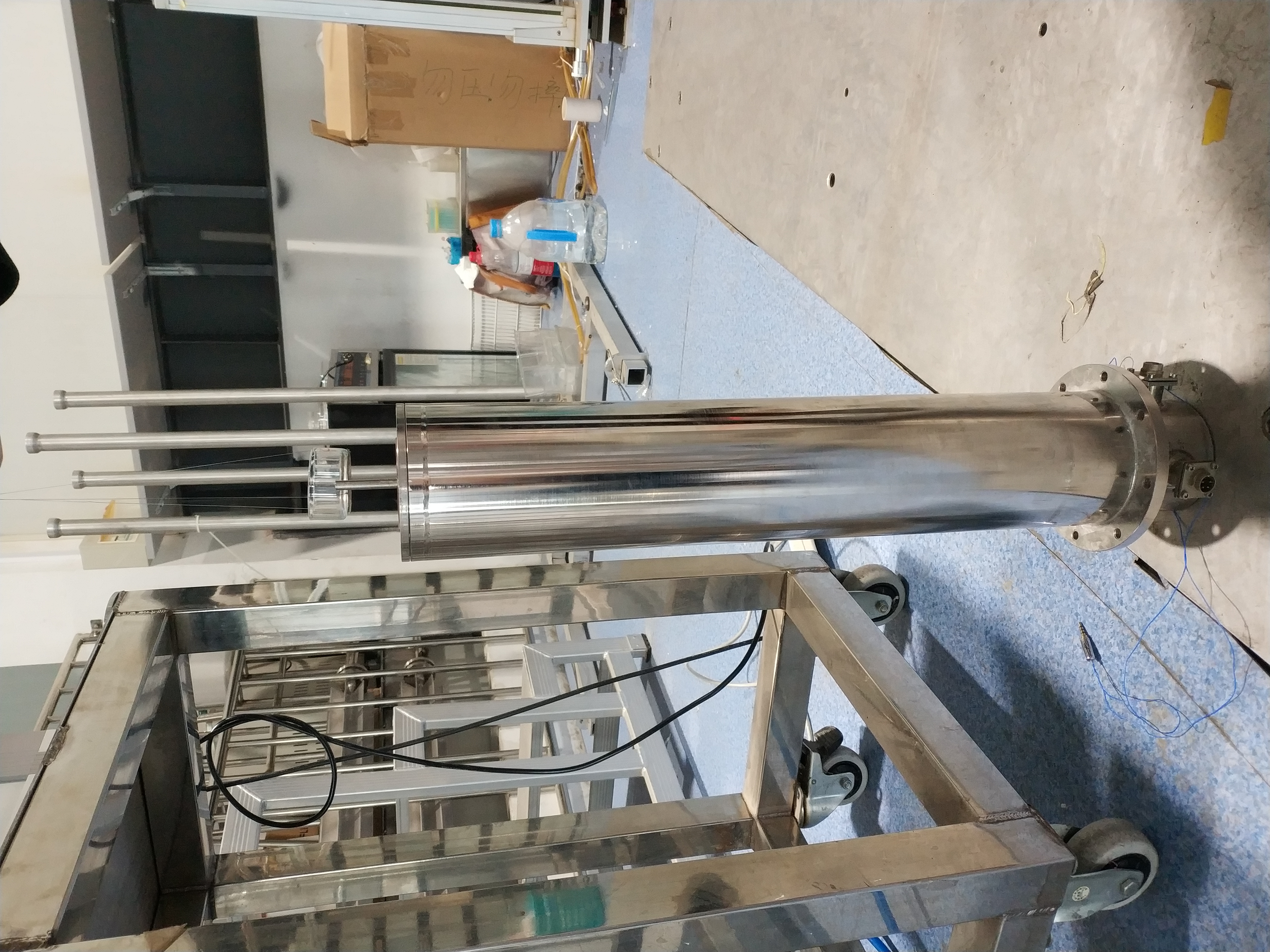

Supplement: Original data [file rsob190137supp1.zip › original data for RSOB-19-0137/Figure 8 superconducting magnet/IMG_20180414_104159_1.jpg]

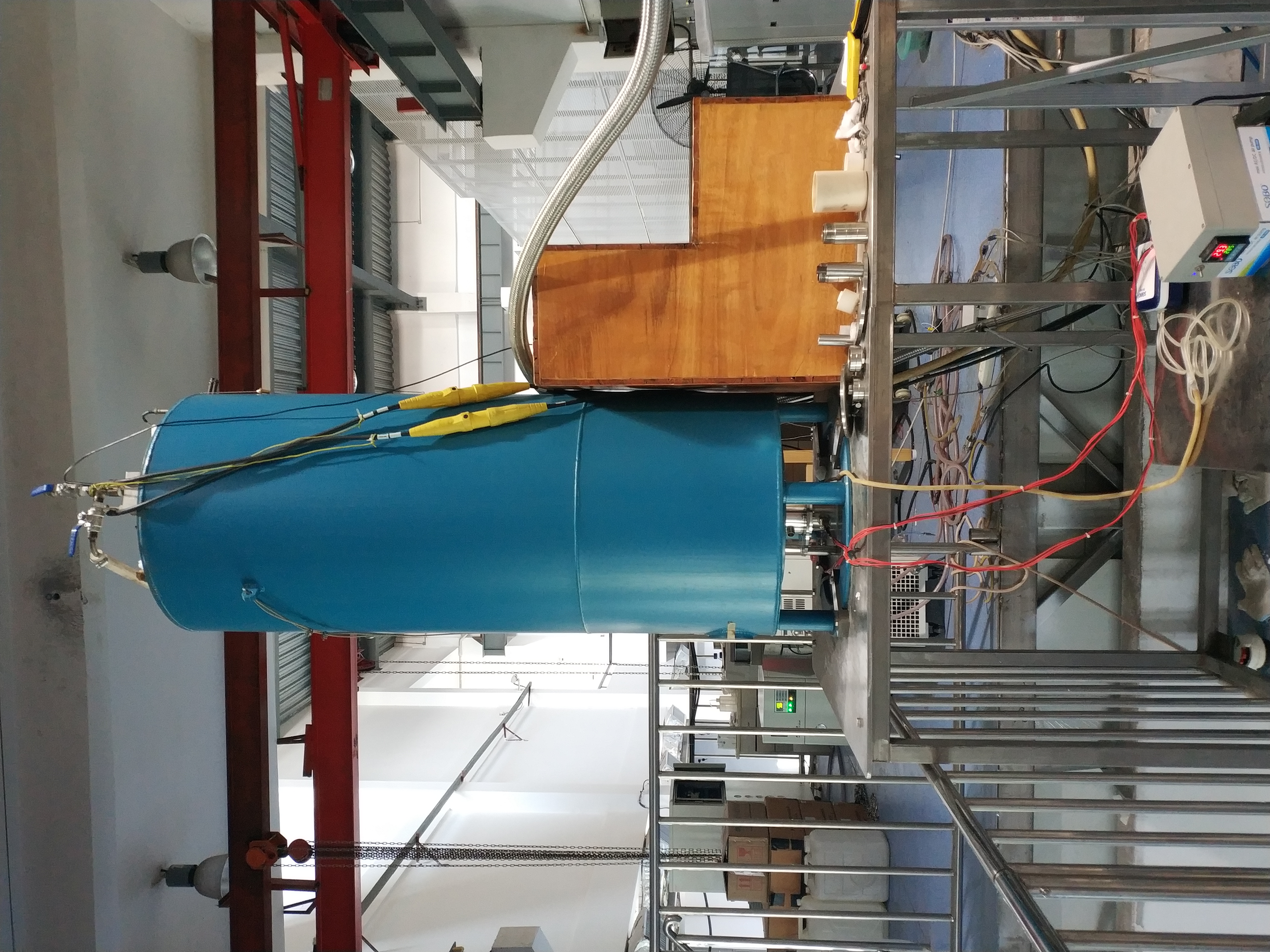

Supplement: Original data [file rsob190137supp1.zip › original data for RSOB-19-0137/Figure 8 superconducting magnet/IMG_20190820_101155.jpg]

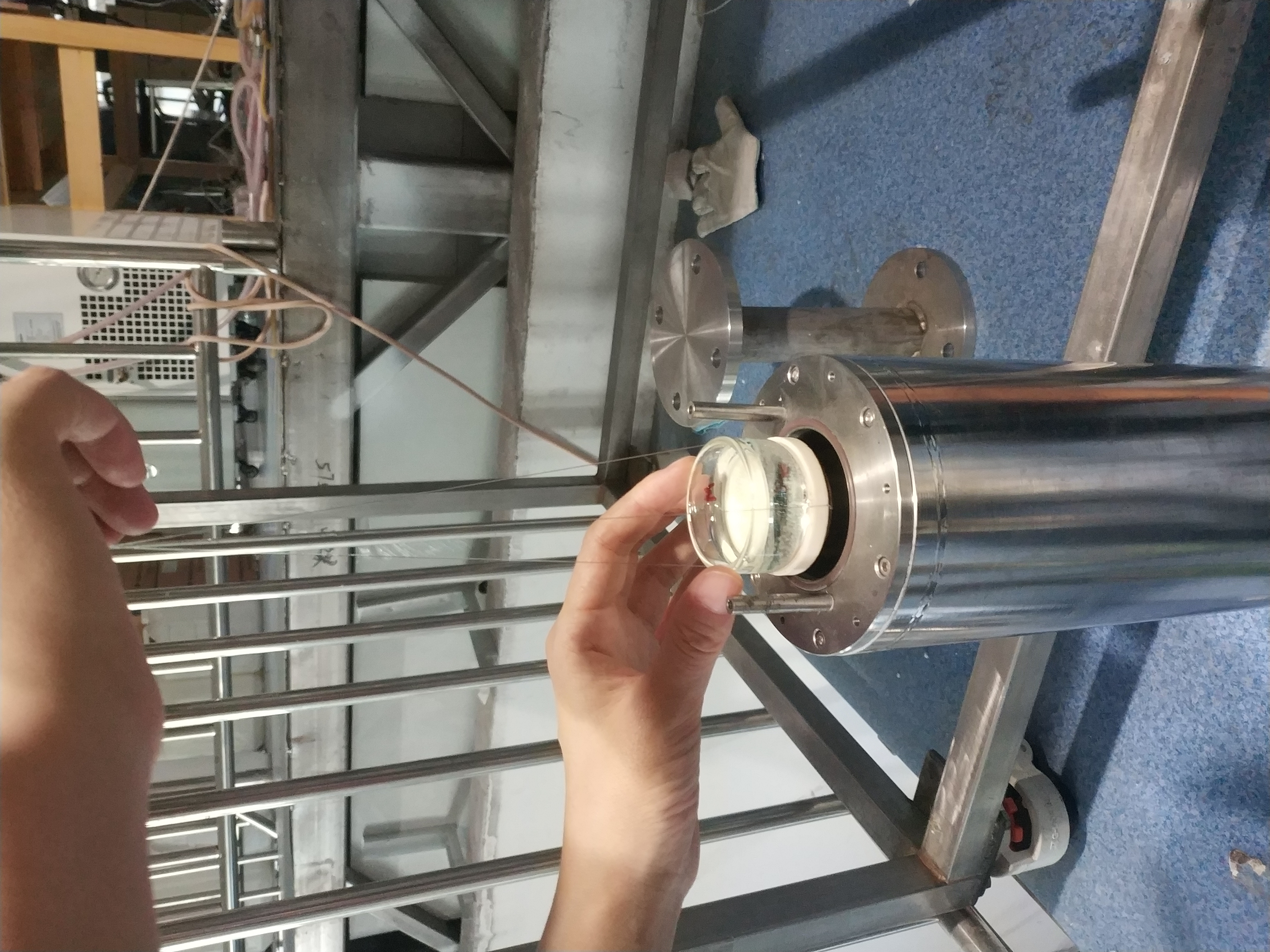

Supplement: Original data [file rsob190137supp1.zip › original data for RSOB-19-0137/Figure 8 superconducting magnet/IMG_20190820_095047.jpg]

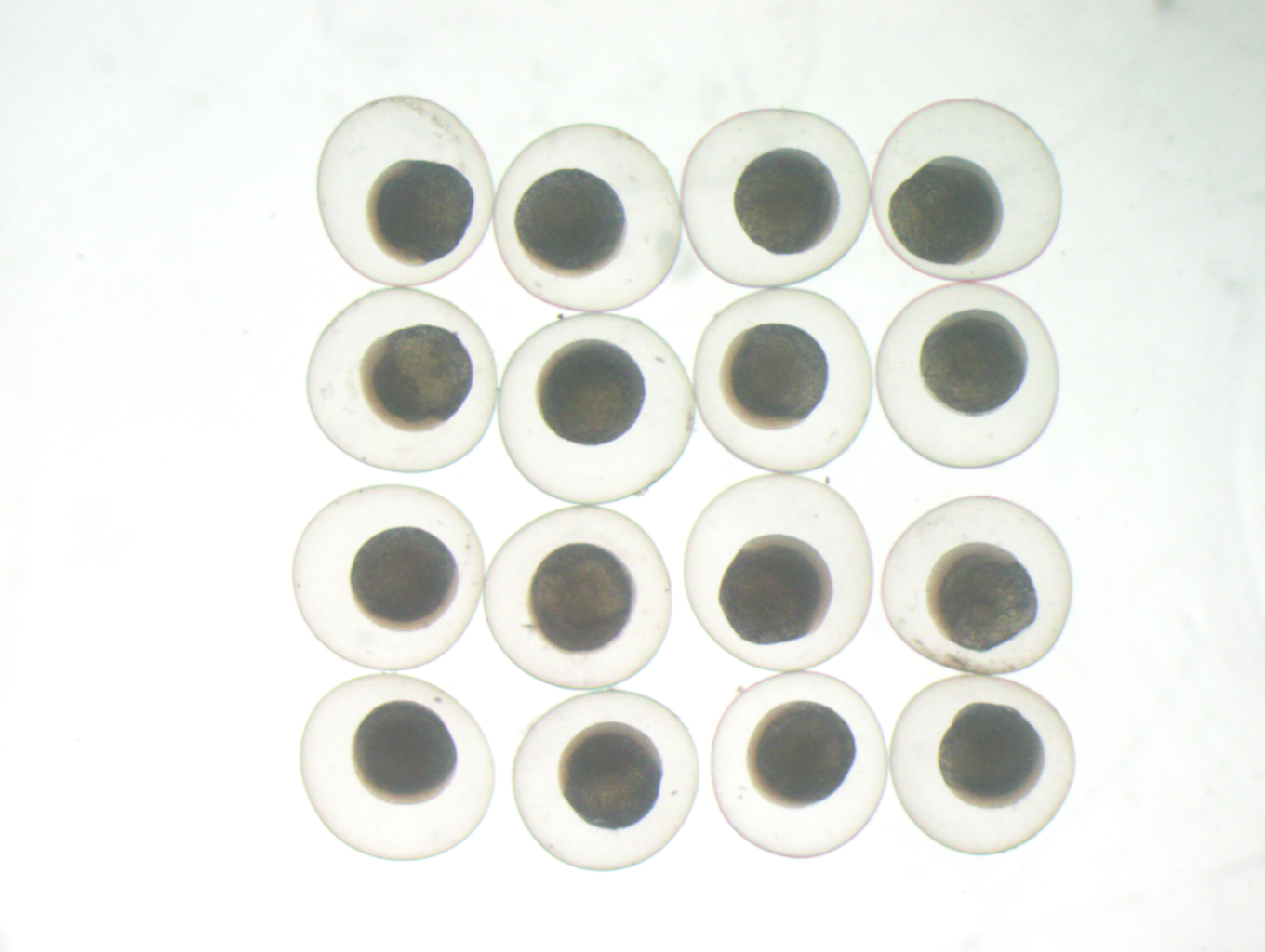

Supplement: Original data [file rsob190137supp1.zip › original data for RSOB-19-0137/Figure 1 snapshots original data/4 hpf/C-4H-1-1.6X.tif]

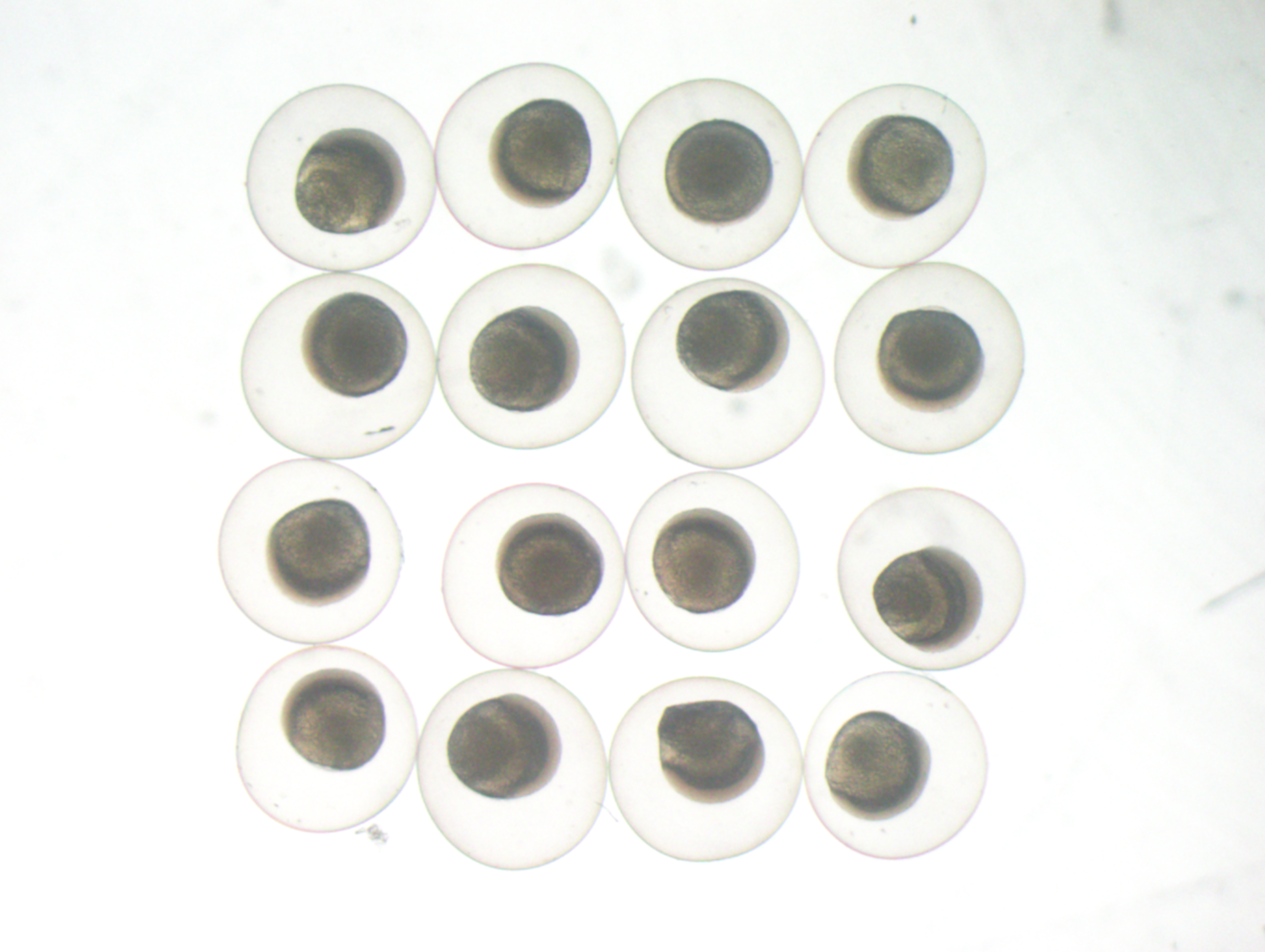

Supplement: Original data [file rsob190137supp1.zip › original data for RSOB-19-0137/Figure 1 snapshots original data/4 hpf/M-4H-2-1.6X.tif]

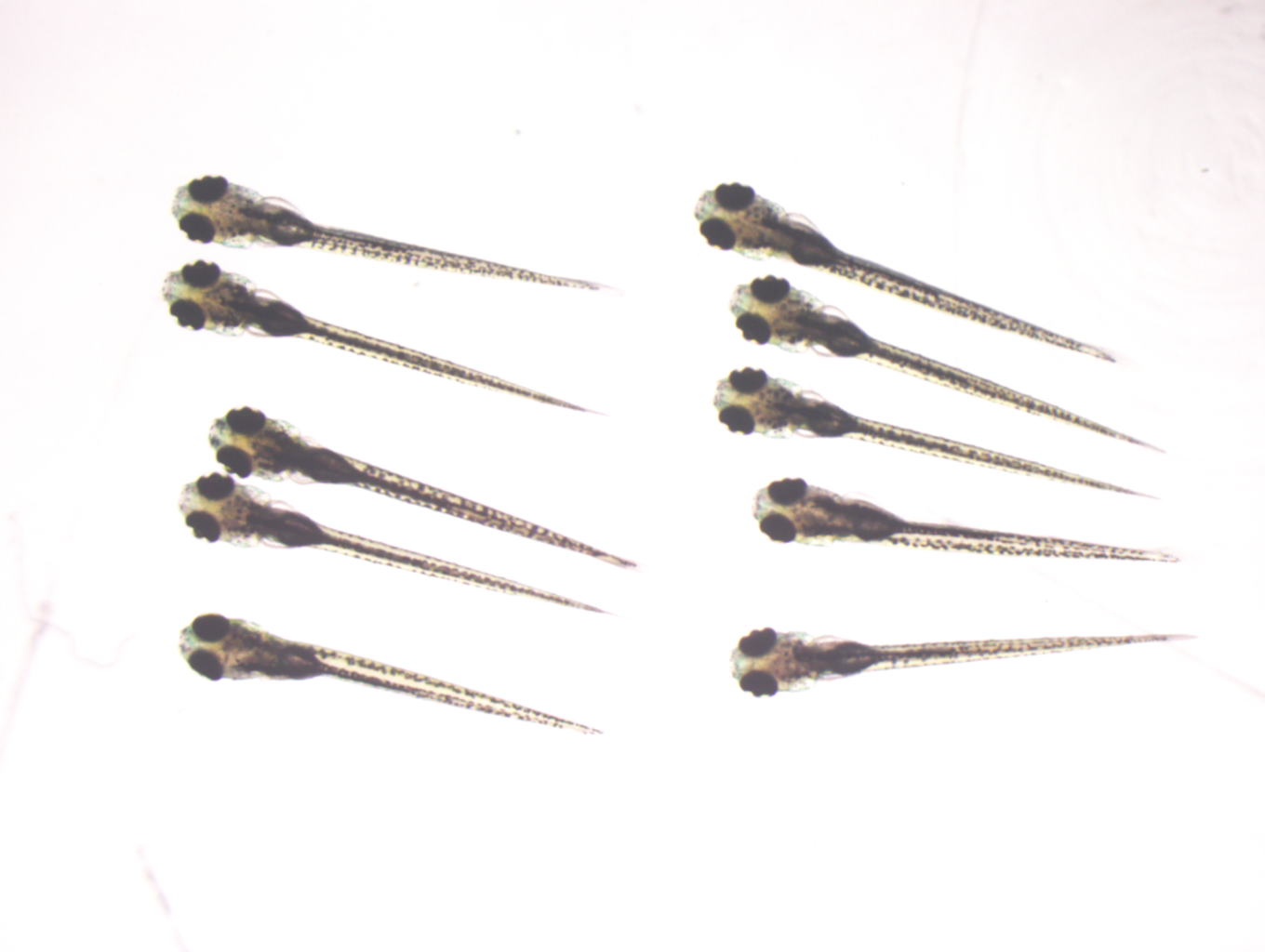

Supplement: Original data [file rsob190137supp1.zip › original data for RSOB-19-0137/Figure 1 snapshots original data/6 dpf/C 1.25X 01-used.tif]

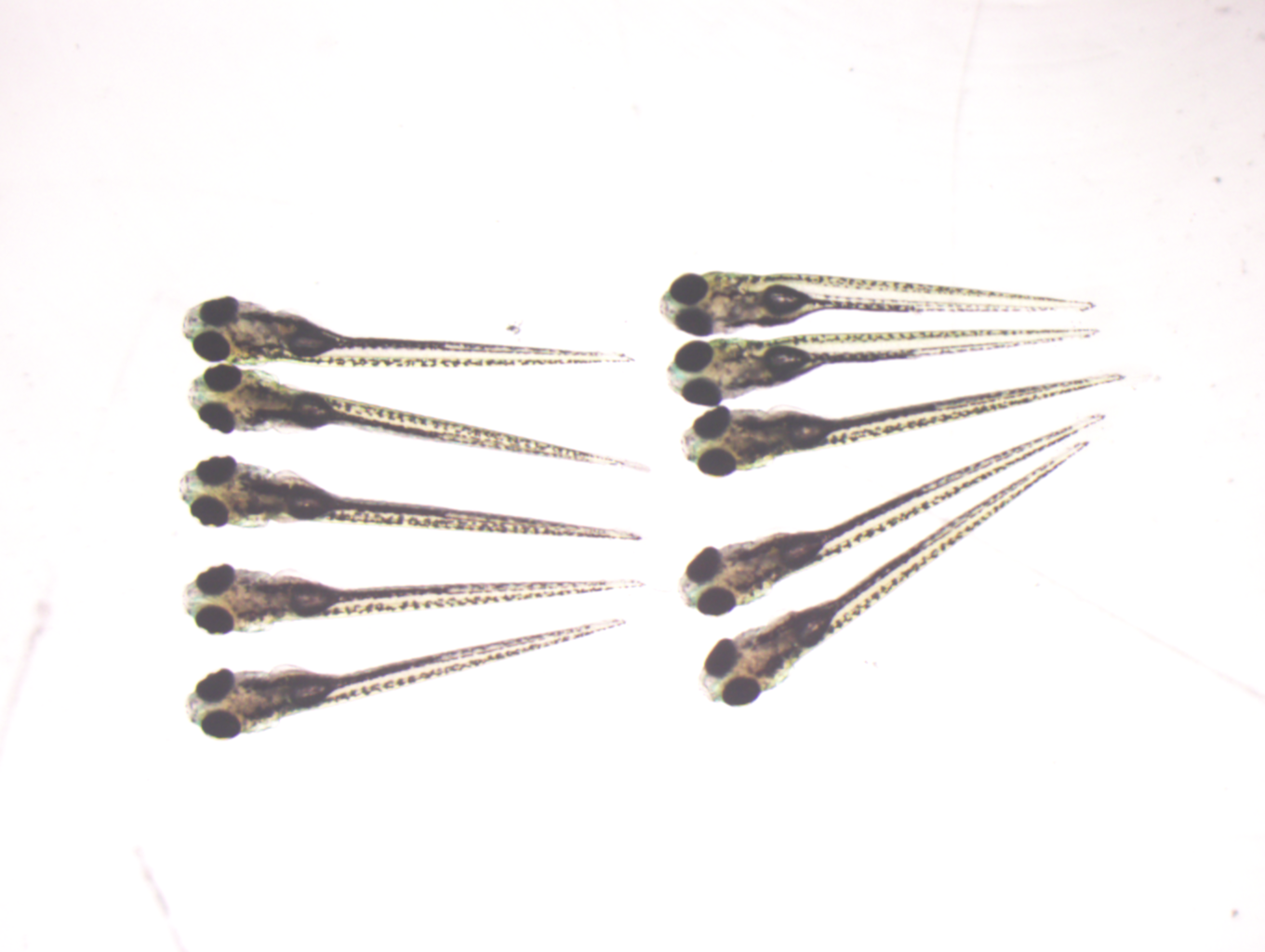

Supplement: Original data [file rsob190137supp1.zip › original data for RSOB-19-0137/Figure 1 snapshots original data/6 dpf/M 1.25X 03-used.tif]

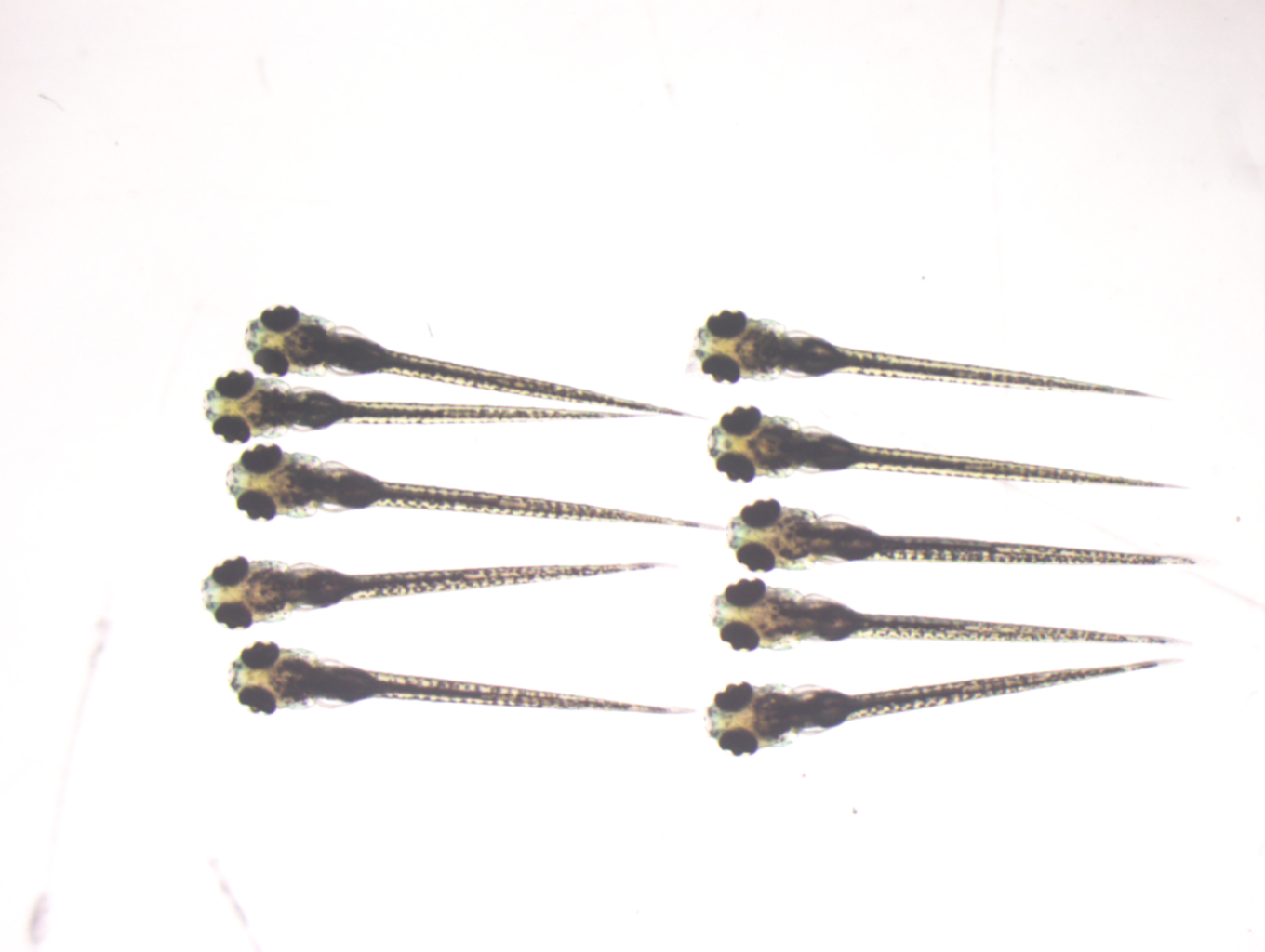

Supplement: Original data [file rsob190137supp1.zip › original data for RSOB-19-0137/Figure 1 snapshots original data/5 dpf/M 1.25X 01-used.tif]

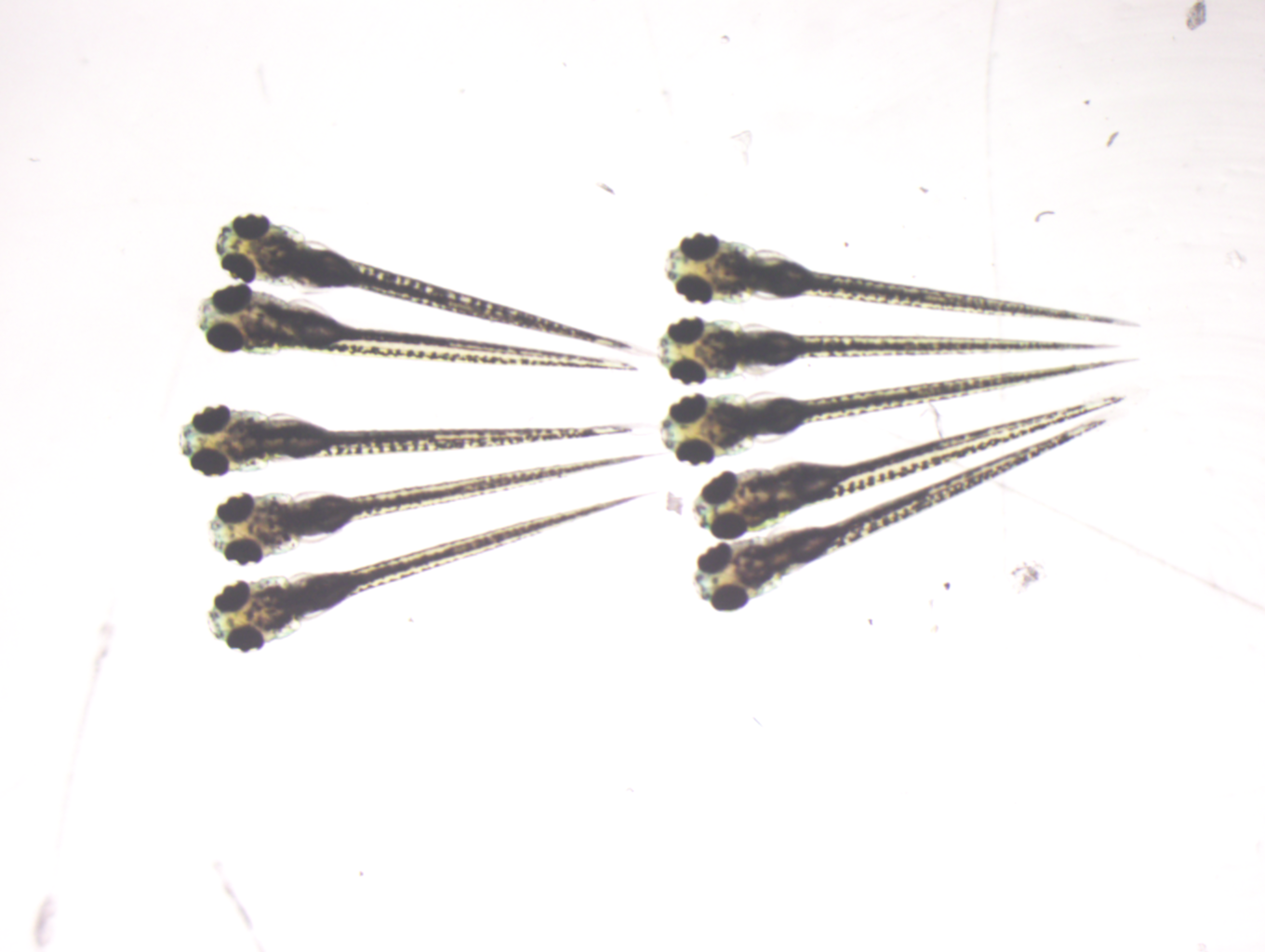

Supplement: Original data [file rsob190137supp1.zip › original data for RSOB-19-0137/Figure 1 snapshots original data/5 dpf/C 1.25X 03-used.tif]

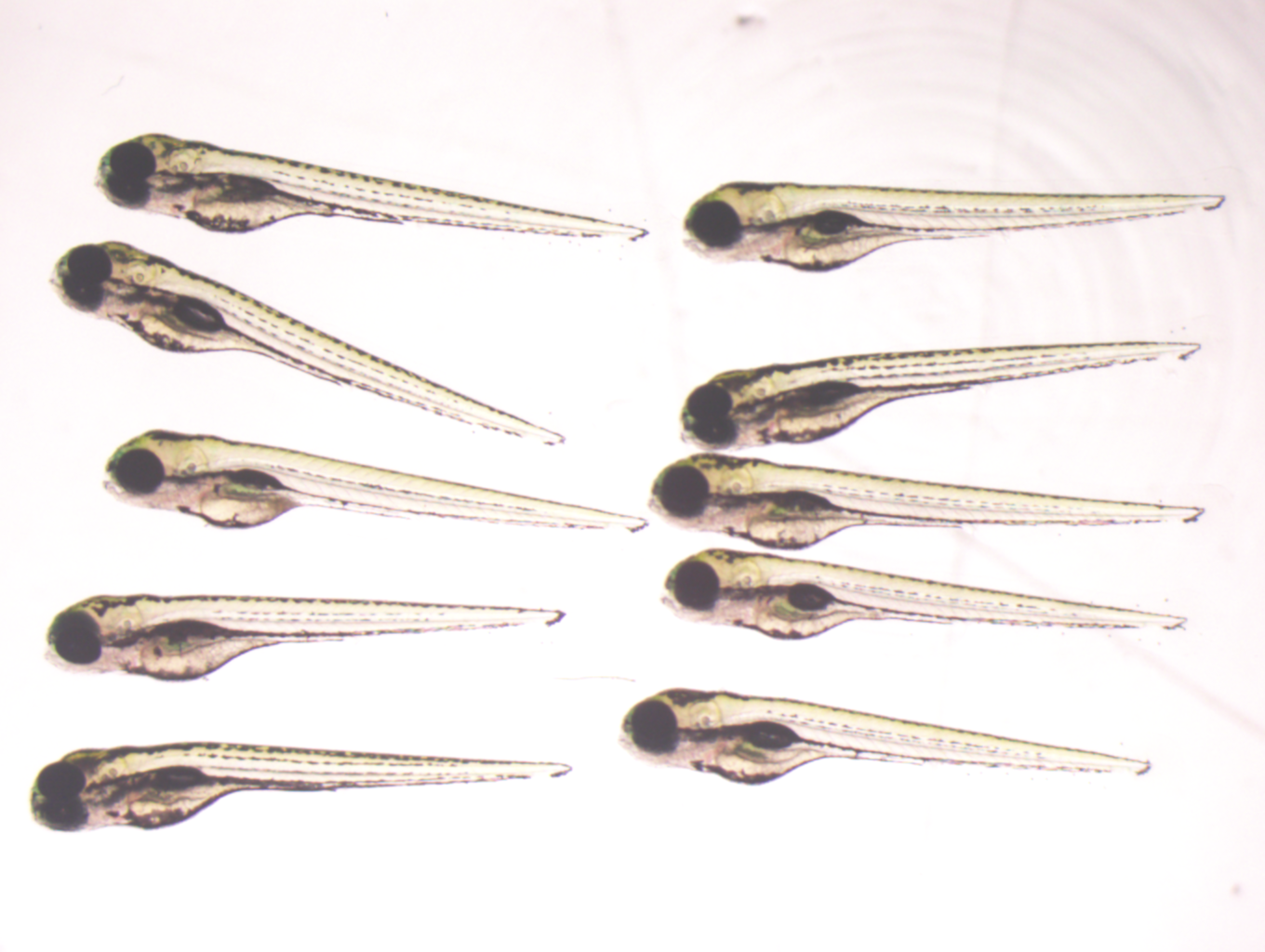

Supplement: Original data [file rsob190137supp1.zip › original data for RSOB-19-0137/Figure 1 snapshots original data/4 dpf/M 1.6X 01-used.tif]

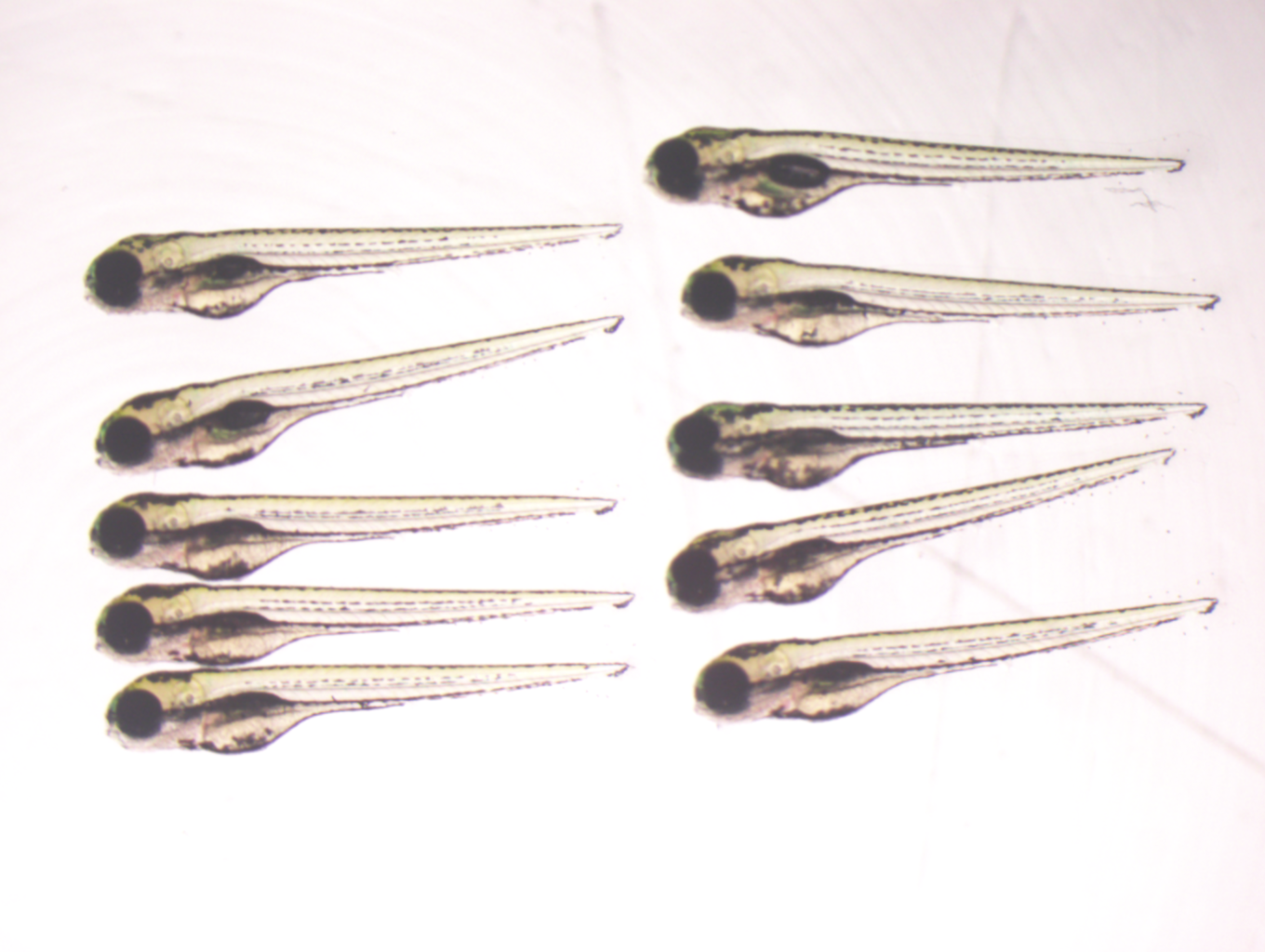

Supplement: Original data [file rsob190137supp1.zip › original data for RSOB-19-0137/Figure 1 snapshots original data/4 dpf/C 1.6X 03-used.tif]

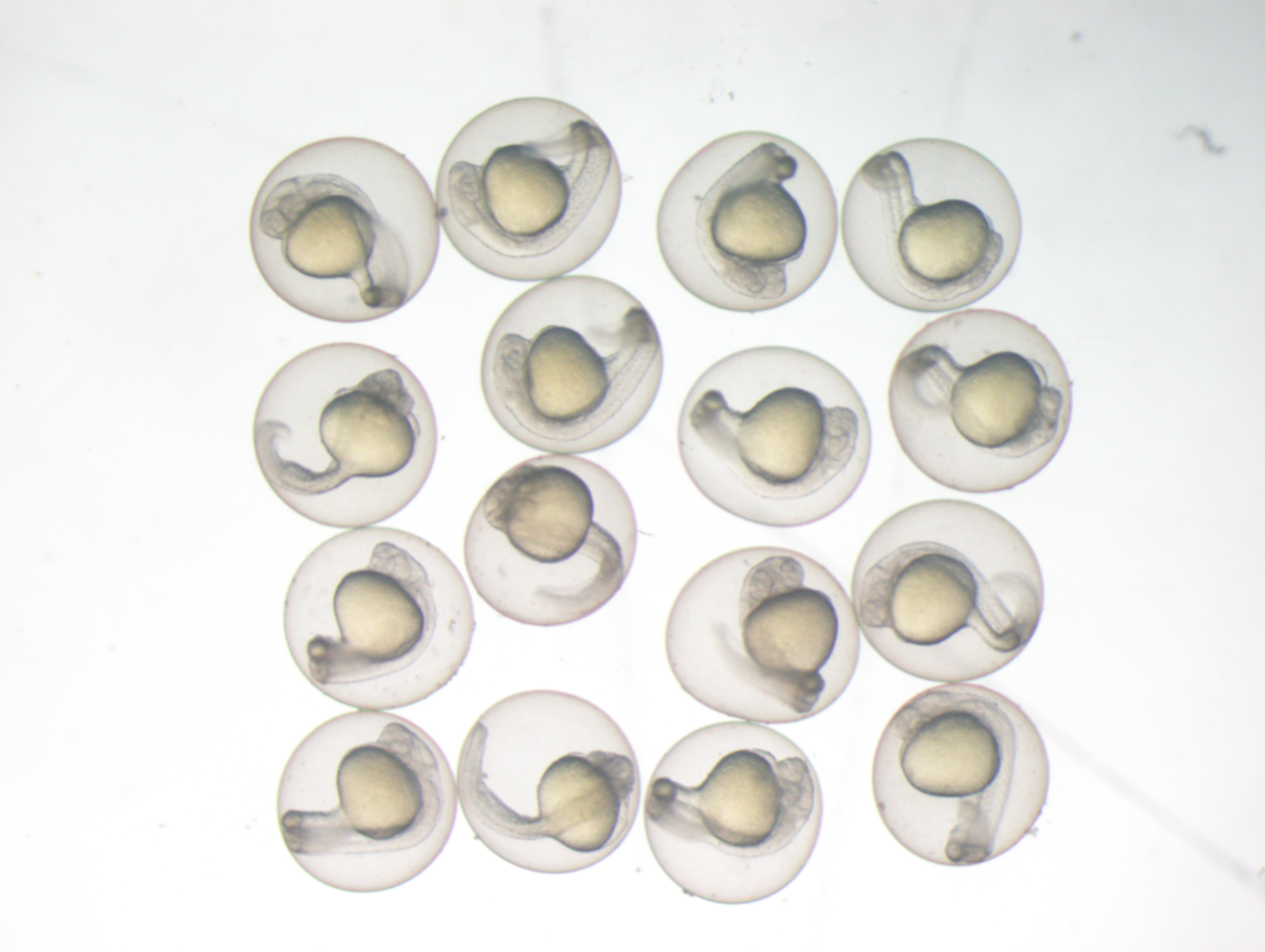

Supplement: Original data [file rsob190137supp1.zip › original data for RSOB-19-0137/Figure 1 snapshots original data/1 dpf/C 1.6X 01-used.tif]

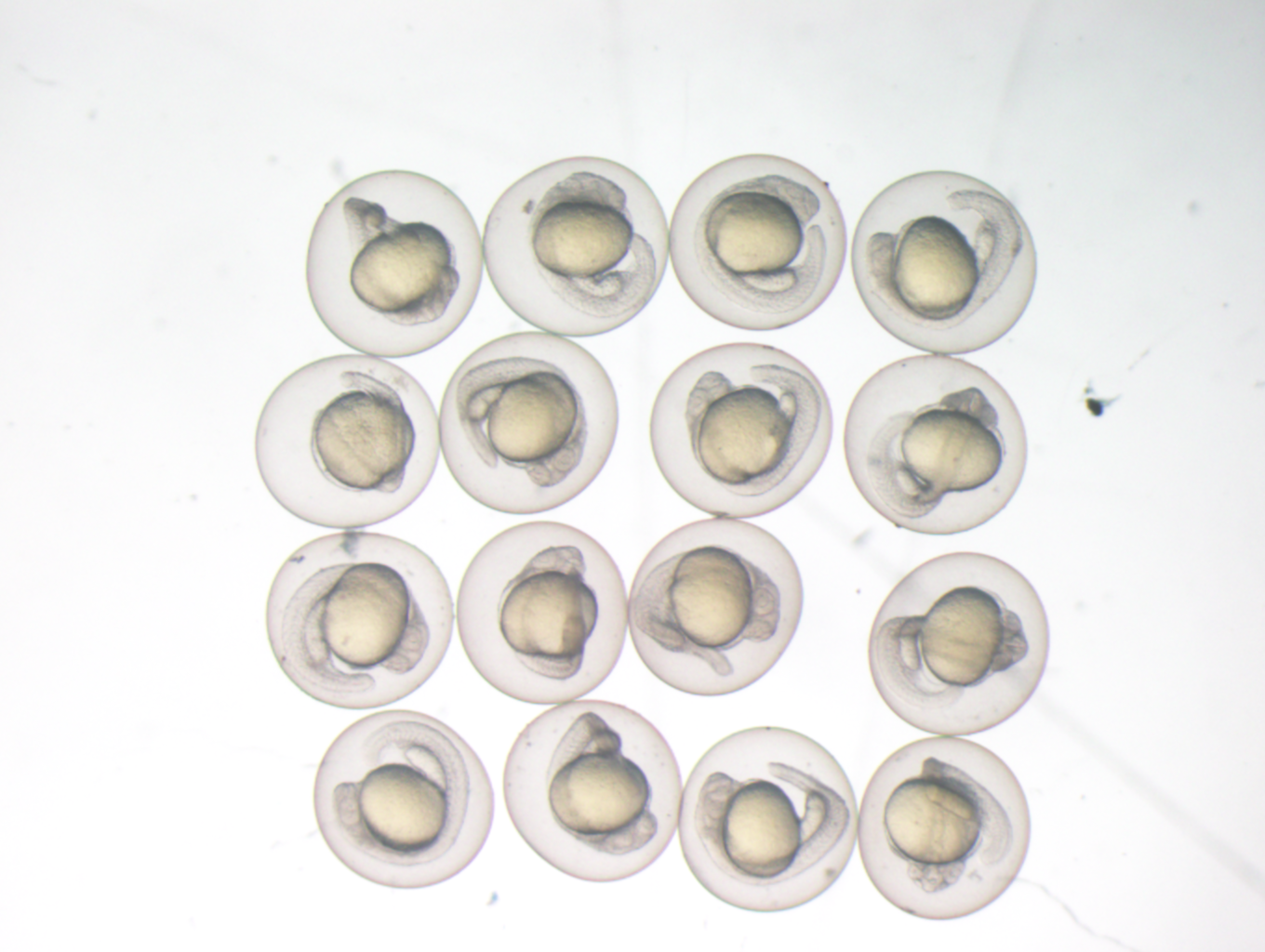

Supplement: Original data [file rsob190137supp1.zip › original data for RSOB-19-0137/Figure 1 snapshots original data/1 dpf/M 1.6X 05-used.tif]

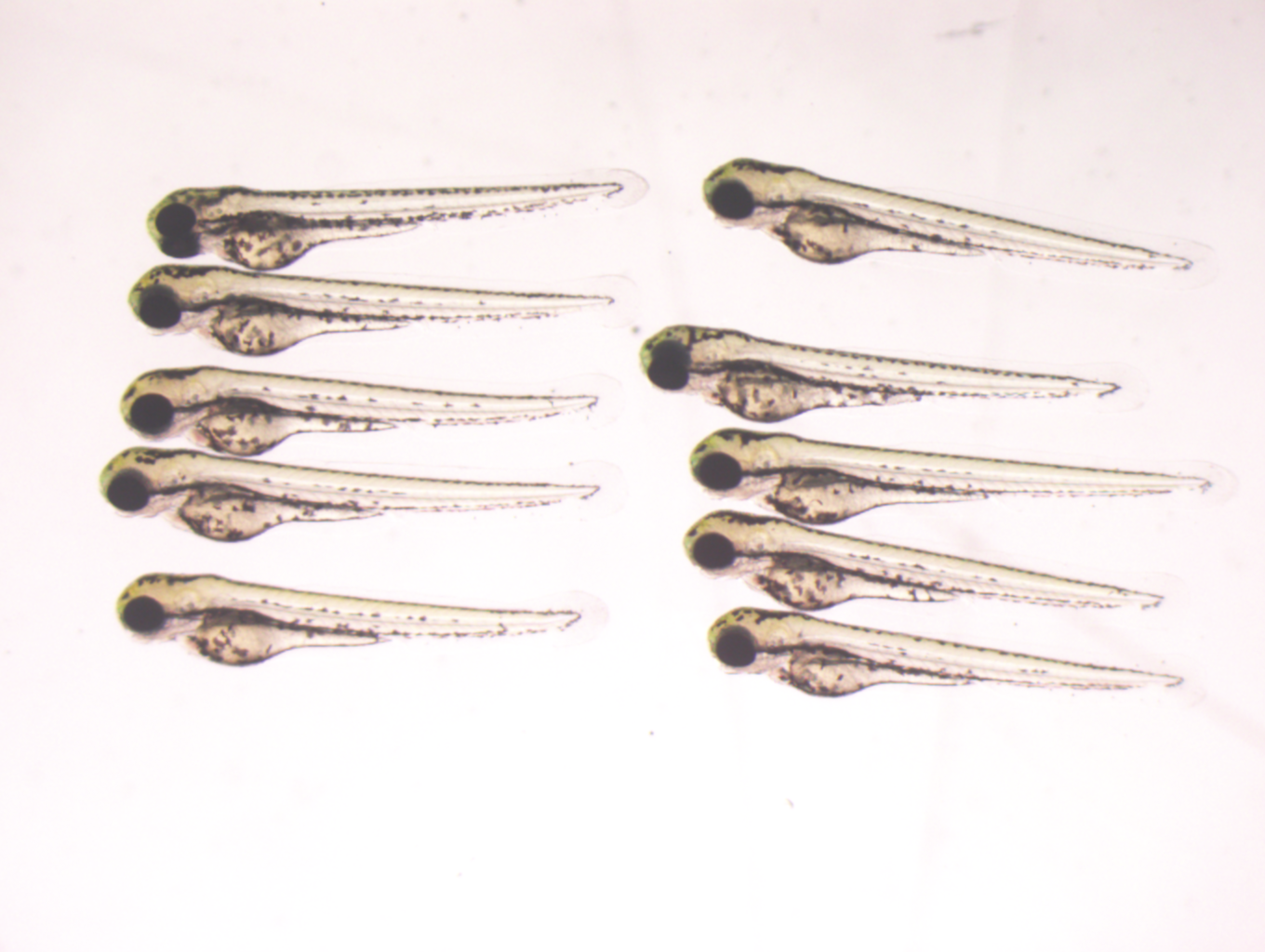

Supplement: Original data [file rsob190137supp1.zip › original data for RSOB-19-0137/Figure 1 snapshots original data/3 dpf/M 1.6X 03-used.tif]

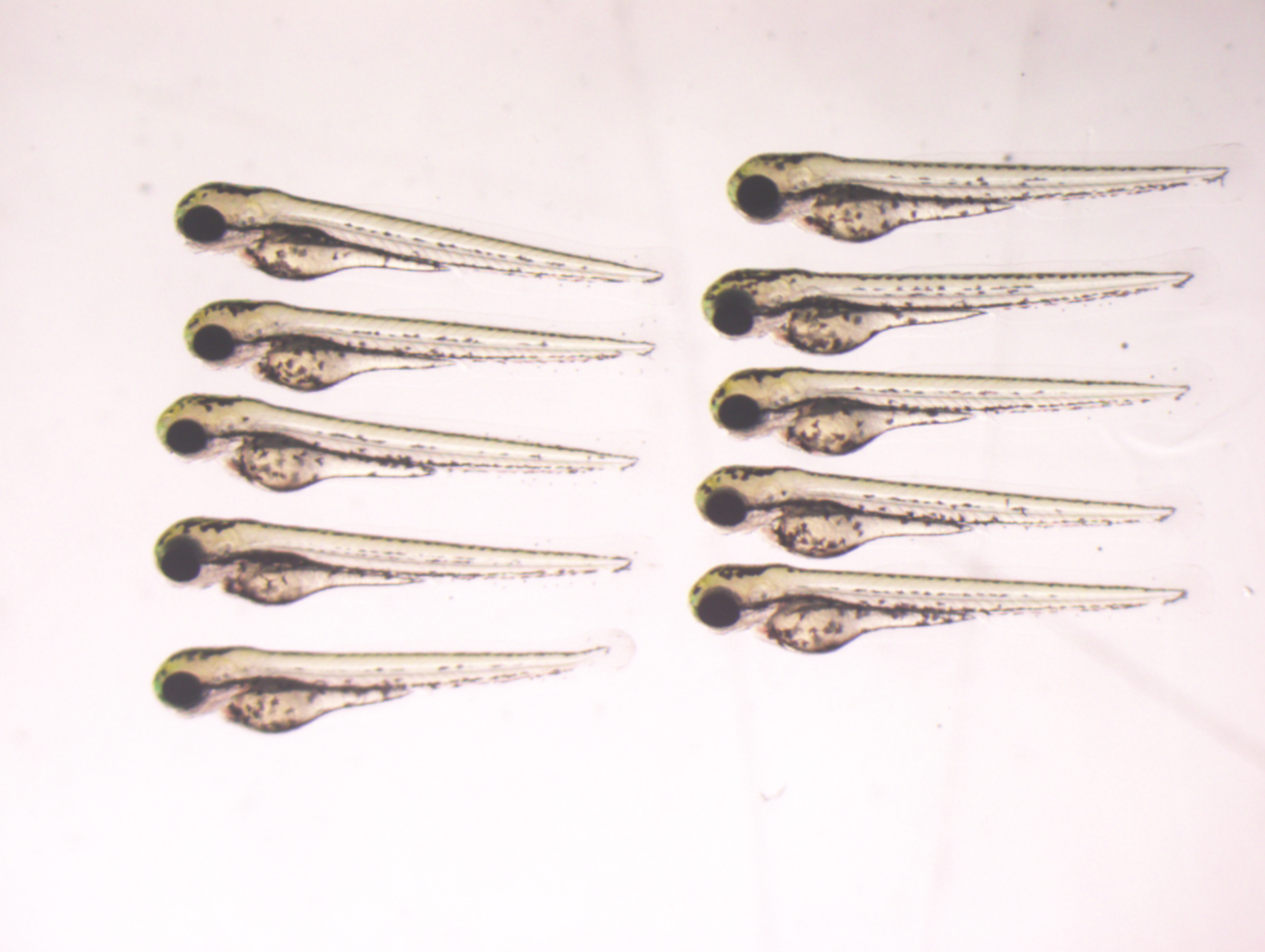

Supplement: Original data [file rsob190137supp1.zip › original data for RSOB-19-0137/Figure 1 snapshots original data/3 dpf/C 1.6X 04-used.tif]

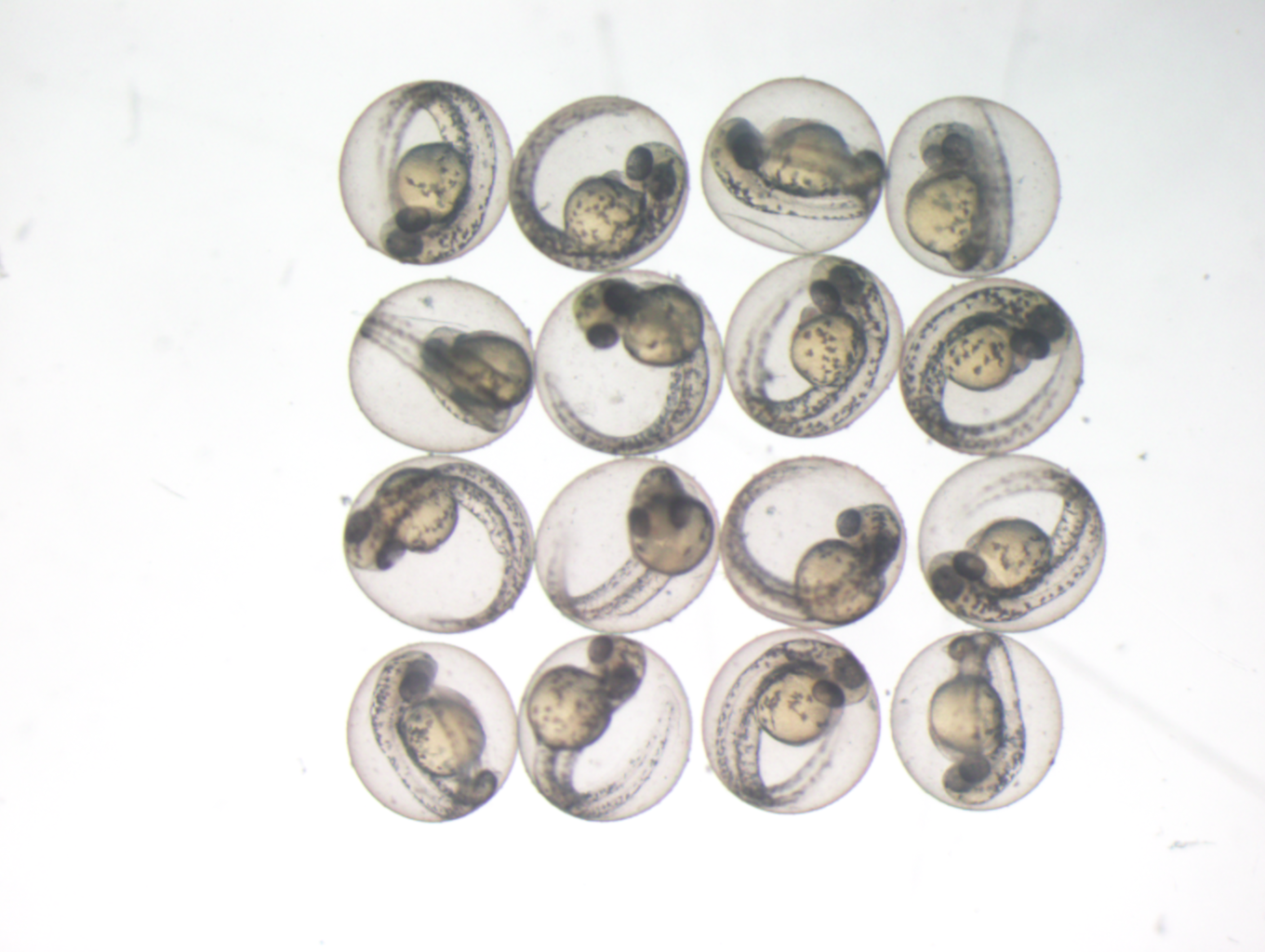

Supplement: Original data [file rsob190137supp1.zip › original data for RSOB-19-0137/Figure 1 snapshots original data/2 dpf/C2 1.6X 01-used.tif]

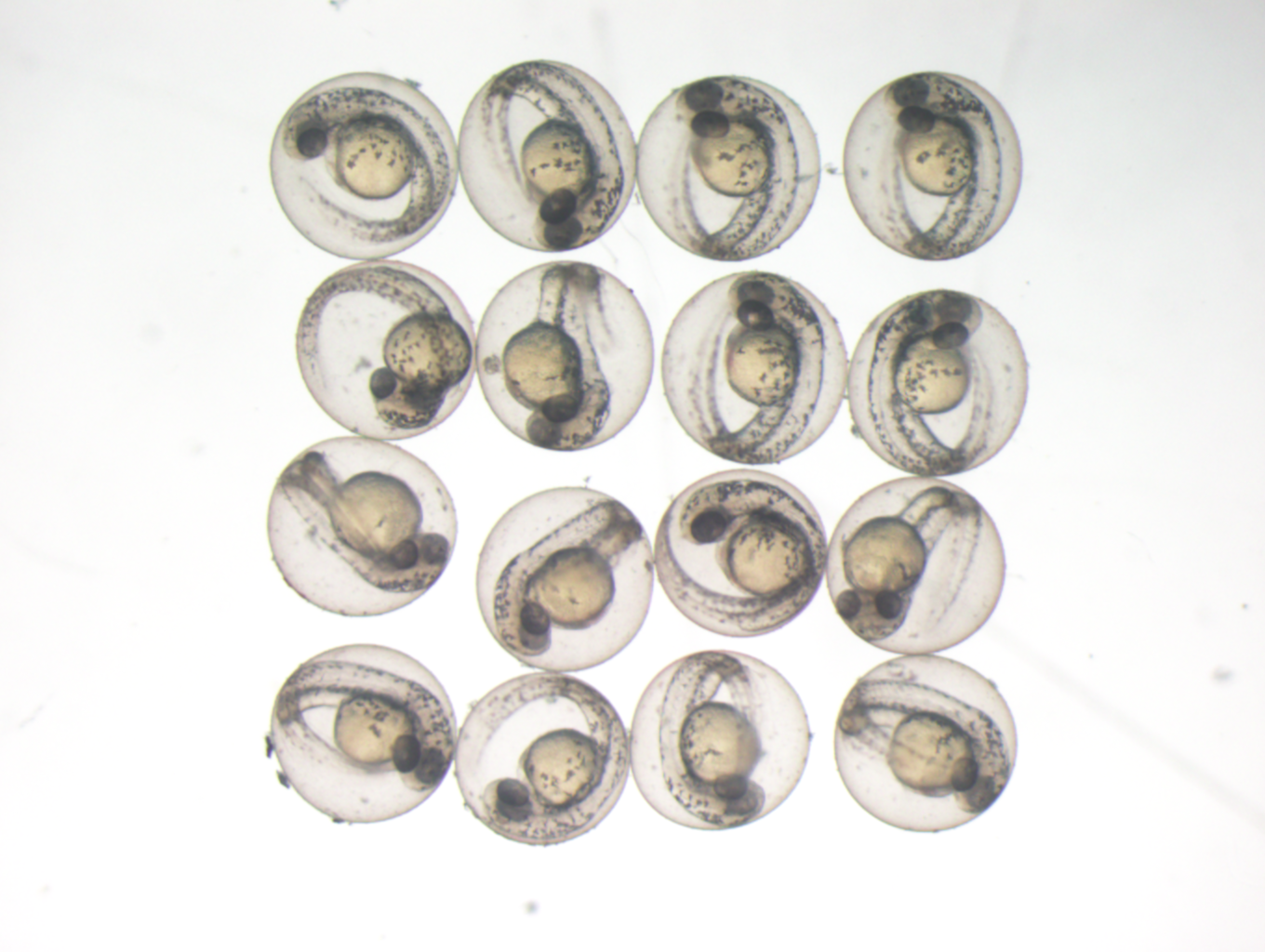

Supplement: Original data [file rsob190137supp1.zip › original data for RSOB-19-0137/Figure 1 snapshots original data/2 dpf/M2 01 1.6X-used.tif]

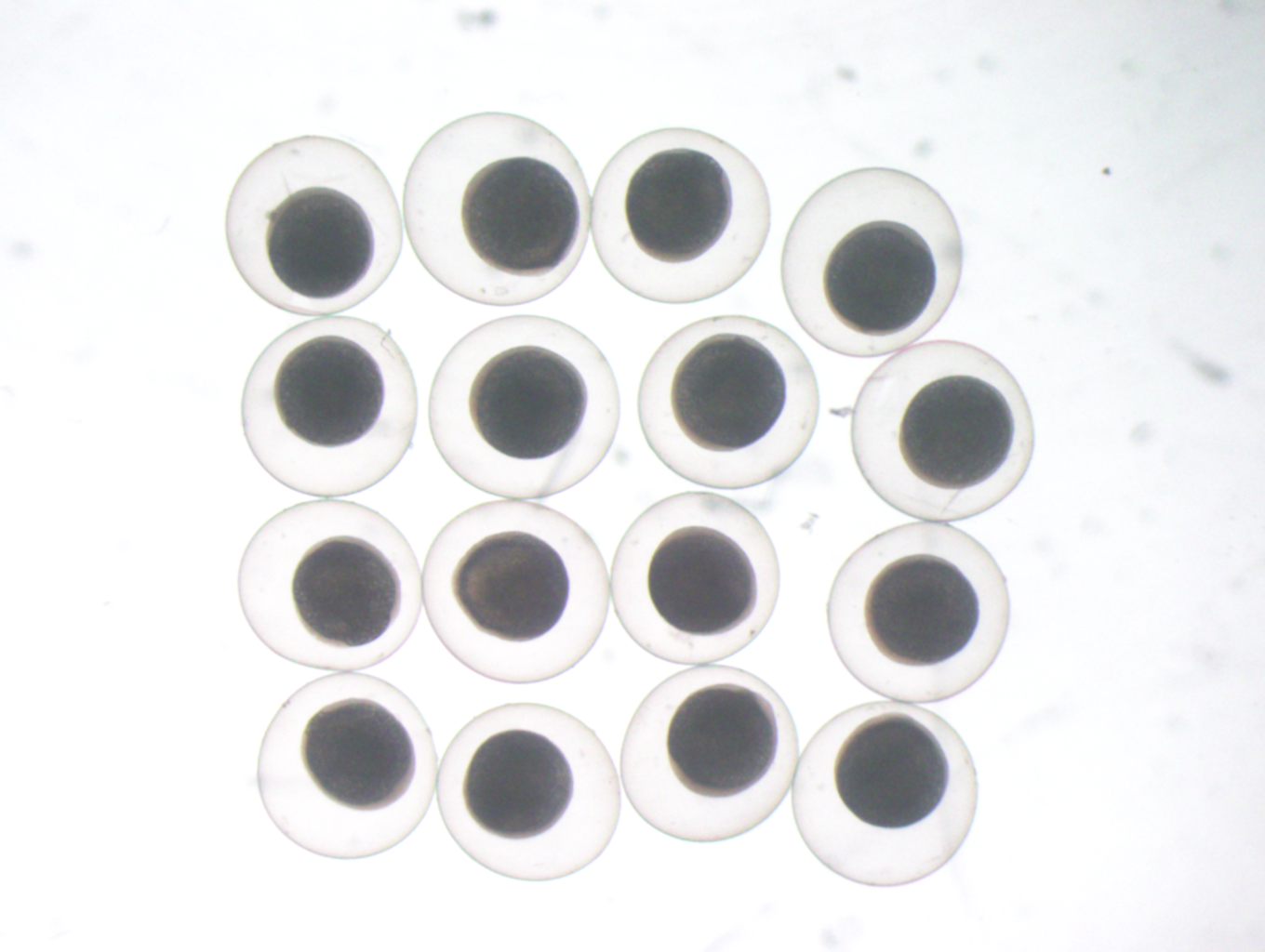

Supplement: Original data [file rsob190137supp1.zip › original data for RSOB-19-0137/Figure 1 snapshots original data/8 hpf/M-8HPF-1.6X-4.tif]

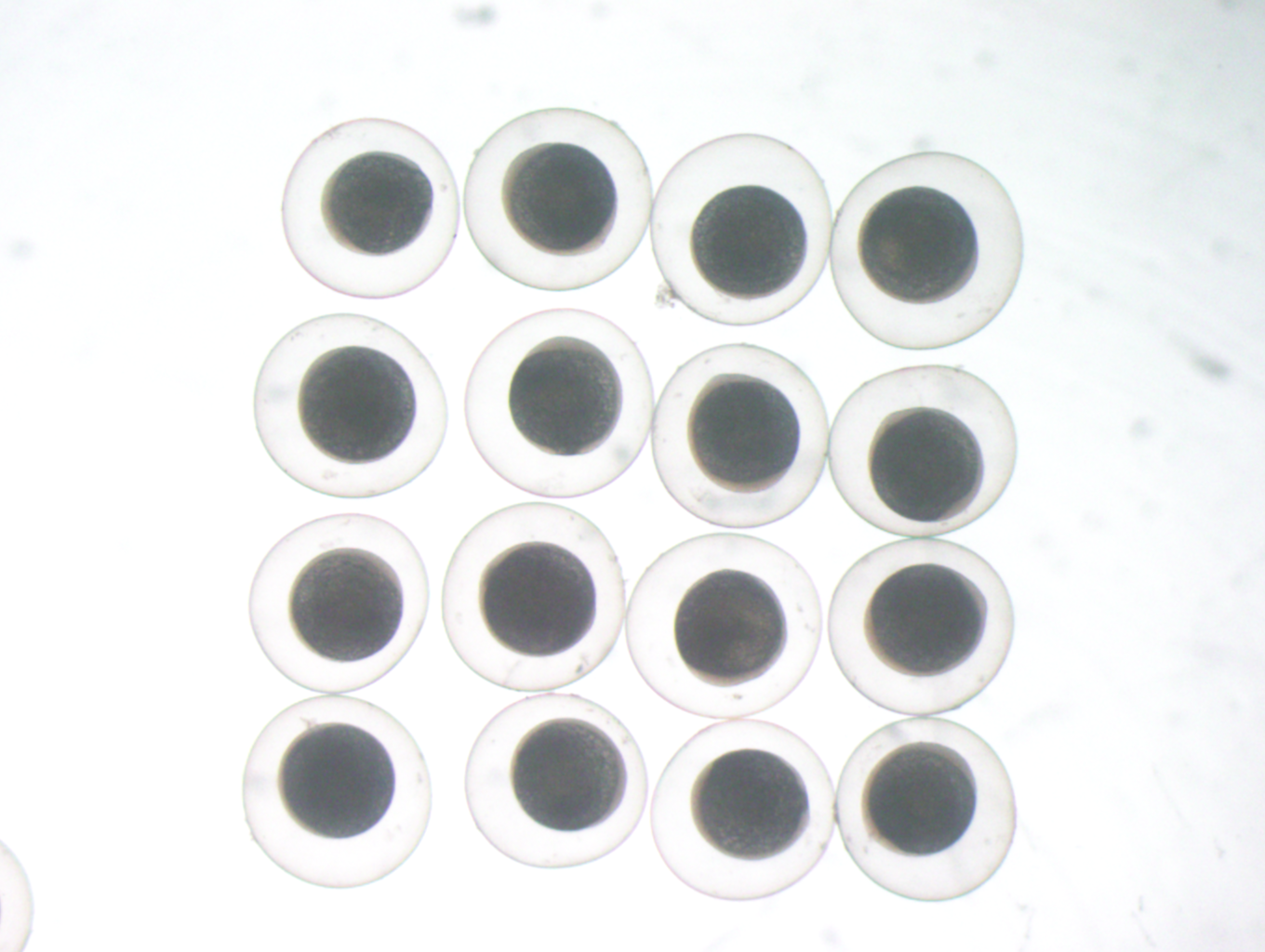

Supplement: Original data [file rsob190137supp1.zip › original data for RSOB-19-0137/Figure 1 snapshots original data/8 hpf/C-8HPF-1.6X-2.tif]
